# CARTOON-based educational intervention for children to foster hygiene knowledge and emotional resilience in preschool children: a randomized controlled trial

**DOI:** 10.3389/fped.2025.1514793

**Published:** 2025-04-11

**Authors:** Philipp Steinbauer, Magdalena Bichler, Renate Fuiko, Sanja Seferagic, Hanna Haas, Tamara Lisy, Sophie Weinmüller, Angelika Berger, Monika Olischar, Agnes Panagl, Vito Giordano

**Affiliations:** ^1^ Division of Neonatology, Pediatric Intensive Care and Neuropediatrics, Department of Pediatrics, Comprehensive Center for Pediatrics Medical University of Vienna, Vienna, Austria; ^2^ Department of Child and Adolescent Psychiatry, Comprehensive Center for Pediatrics, Medical University of Vienna, Vienna, Austria

**Keywords:** child well-being, hygiene-education, anxiety, RCT – randomized controlled trial, psycho-educational, hygiene

## Abstract

**Background:**

In the last four years, COVID-19 has prompted concerns about children's well-being. While children's physical health may not be severely affected, their psychological well-being is a significant concern. Therefore, we developed an interdisciplinary psychoeducational intervention program for children named CARTOON. The study aimed to assess whether CARTOON helps kindergarten children to better adhere to mandatory hygiene measures, while also investigating its potential to reduce children's anxiety.

**Methods:**

A randomized controlled trial was conducted from January and June 2021 in two Viennese kindergartens involving 53 children aged 3–6 years. Children were randomized into an intervention (specific psychoeducational program) and a control group (routine hygiene practices). CARTOON comprised five sessions covering key hygiene and COVID-19 awareness aspects, facilitated by trained staff. Primary outcomes included changes in children's emotional state and knowledge about COVID-19 and hygiene measures. Secondary outcomes assessed CARTOON's long-term impact on knowledge retention and emotional well-being.

**Results:**

Children in the intervention group showed increased COVID-19 knowledge from baseline to long-term assessment (3.9 ± 2.4 vs. 5.9 ± 2.0, *p* = .003). Moreover, children in the intervention group demonstrated significantly improved long-term hygiene knowledge compared to baseline (8.2 ± 2.2 vs. 5.9 ± 2.3, *p* < 0.001). Additionally, anxiety scores significantly decreased in the intervention group post-intervention compared to baseline (2.2 ± 1.7 vs. 5.6 ± 2.5; *p* = .01). Regression analysis identified higher age, higher maternal education, and lower family mental stress as knowledge acquisition predictors.

**Conclusions:**

Our findings demonstrate the efficacy of our psycho-educational program in enhancing both knowledge about COVID-19 and adherence to hygiene measures among preschool children, while also reducing anxiety related to the pandemic.

**Clinical Trial Registration:**

https://clinicaltrials.gov/ct2/show/NCT04724616.

## 1 Introduction

Pandemics have historically marked significant junctures in the course of human history, and the ongoing global spread of the COVID-19 pandemic, caused by severe acute respiratory syndrome coronavirus 2 (SARS-CoV-2), underscores the need for proactive measures to address the multifaceted impact on society. While current evidence suggests that children tend to experience milder physical health effects from COVID-19, urgent inquiries are required to comprehend the transmission dynamics and specific risks associated with children (1–3).

Beyond the immediate health implications, the widespread economic and social disruptions resulting from the pandemic pose a substantial risk to the mental health of children. Numerous studies have documented a surge in moderate-to-severe symptoms of anxiety and depression among children and adolescents during the COVID-19 outbreak (4–6). The general threat of the virus, adherence to preventive measures, and routine medical assessments contribute to heightened stress, fostering feelings of fear, depression, and insecurity among young individuals (7, 8).

When the pandemic started, unfolding in waves with unpredictable ends, both parents and educators had to adapt their approaches to imparting knowledge about COVID-19 to children. Preparing for potential restrictions in daily life and instilling protective measures such as mask-wearing, hand hygiene, avoiding face touching, proper sneezing etiquette, and social distancing. Hence, developing effective educational methods would be crucial to navigate potential abrupt changes in daily life, and would equip parents, educators, and children with a communication tool also useful for future events.

In response to this need, drawing inspiration from psycho-educational patient training courses developed for chronic diseases (9), we emphasize the significance of psycho-educational interventions in pediatric care (10). Research indicates that targeted information and the acquisition of coping strategies can alleviate stress and fears, with potential applications in the pediatric context (11). Notably, studies have revealed that children and adolescents often exhibit suboptimal preventive practices compared to adults (12). Cartoon-based interventions have been shown to be highly effective in delivering educational content to young children. They simplify complex concepts, engage children's attention, and enhance retention through visual storytelling and repetition. Studies have demonstrated that animated cartoons significantly improve health-related behaviors in children (13–15). These studies collectively suggest that cartoon-based interventions can offer significant advantages over traditional educational methodologies, including increased engagement, better knowledge retention, and more sustainable behavior change, making them a valuable tool in children's hygiene education (13, 15). Given the effectiveness of this approach, we specifically have developed an interdisciplinary psycho-educational intervention program tailored for children in kindergarten, titled CARTOON (CoronAviRus educaTional prOgram fOr childreN). This initiative aims not only to address the immediate impact of the current pandemic but also to establish a valuable resource for future events, fostering resilience and preparedness among children, parents, and educators alike.

Furthermore, enhancing hygienic knowledge within this age group holds promise not only for managing the current pandemic but also for addressing other seasonal illnesses such as influenza or the common cold. By instilling good hygiene practices early on, the CARTOON program seeks to establish a foundation that contributes to overall health and well-being, offering lasting benefits beyond the challenges posed by the coronavirus pandemic.

The implementation of the specialized CoronAviRus educaTional prOgram fOr children (CARTOON) encompassed multiple objectives. Firstly, the study aimed to assess the effectiveness of our educational training program, grounded in cartoon heroes, in enhancing adherence to hygiene measures among preschool children (aged 3–6 years) amid the pandemic. A secondary objective involved the endeavor to bolster children's sense of competence and self-efficacy, with the ultimate goal of mitigating feelings of helplessness and fear. Additionally, the study seeks to evaluate the enduring impact of CARTOON by examining the retention of acquired theoretical and practical knowledge three months post-completion of the educational program.

## 2 Materials and methods

### 2.1 Setting and participants

#### 2.1.1 Study registration

The protocol for our randomized controlled study was pre-registered on clinicaltrials.gov (https://clinicaltrials.gov/ct2/show/NCT04724616).

#### 2.1.2 Study location

This prospective randomized controlled trial took place in two kindergartens located in Vienna, Austria. The first kindergarten comprised four groups, each with 30 children, while the second had three groups, each with 30 children.

#### 2.1.3 Randomization process

Probands were randomized at a 1:1 ratio to the study group (Intervention Group: *n* = 26) or the control arm (Control Group: *n* = 27) by using the Randomizer for Clinical Trials tool developed at the Medical University of Graz (https://www.randomizer.at). To minimize potential confounding effects related to age, participants were stratified into predefined age groups before randomization. Age stratification was performed by categorizing participants into age strata (e.g., 3–4 years, 5–6 years) to ensure a balanced distribution across study groups.

**Figure 1:**
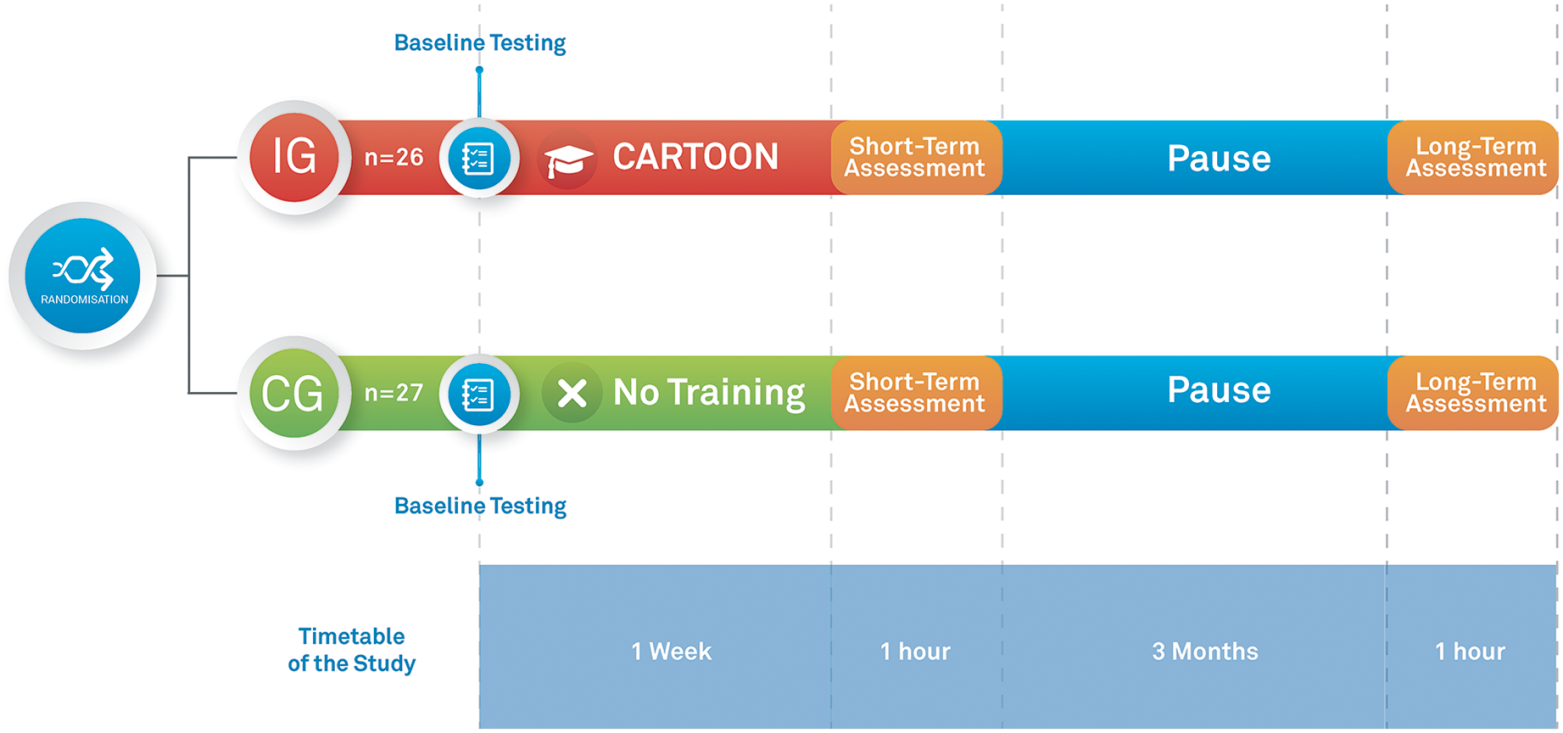
Timetable of the study.

Within each age stratum, participants were randomly assigned to either Group A or Group B in a 1:1 ratio using. Depending on the randomization outcome (intervention group vs. control group), children either underwent the CARTOON psycho-educational training (intervention group) or received no specific training (control group) (Figure 1). Children assigned to the control group did not participate in the structured CARTOON psychoeducational intervention; instead, they continued receiving only the routine hygiene education provided by kindergarten teachers, which typically included regular reinforcement of standard practices such as proper handwashing before meals, after restroom use, and following other basic hygiene routines commonly emphasized in early childhood education settings. Both the intervention and control groups underwent assessments before and after the one-week intervention phase. Additionally, assessments for both groups were conducted three months post-intervention to evaluate the impact of CARTOON on emotional well-being and retention of acquired theoretical and practical knowledge (Figure 1).

#### 2.1.4 Preliminary conference

Before commencing the study, a parent-teacher conference was held to disseminate information about CARTOON and address potential queries from teachers and parents.

#### 2.1.5 Inclusion criteria

Children aged three to six years attending kindergarten, whose parents provided consent, were eligible for inclusion. Participants were required to have sufficient German language skills for study inclusion.

#### 2.1.6 Exclusion criteria

Participants unable to complete any of the training exercises due to physical limitations were excluded. Additionally, individuals and parents with poor German language knowledge and proficiency to comprehend the psychoeducational training instructions were excluded from the study.

**Figure 2:**
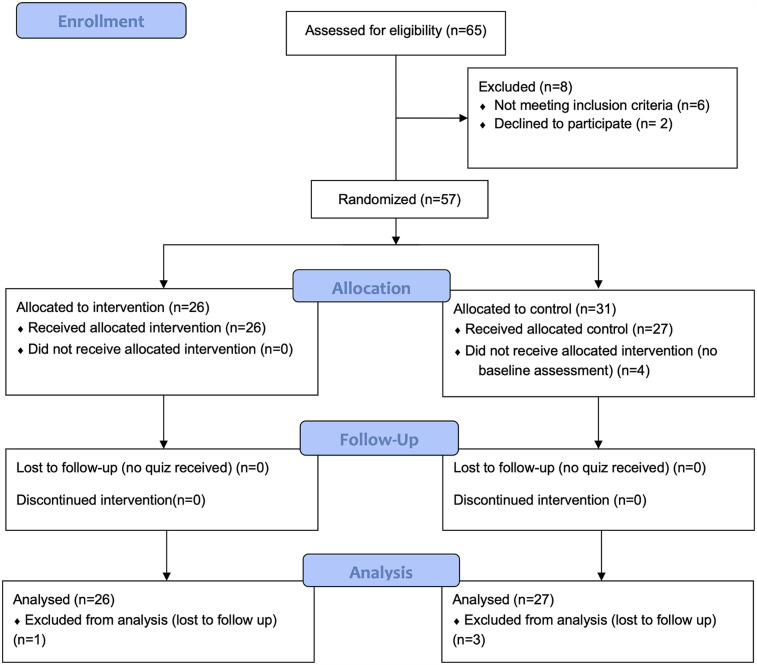
CONSORT flow diagram.

A Consort Flow Diagram is provided in Figure 2.

### 2.2 Coronavirus educational program for children (CARTOON)

Our CARTOON program, an interdisciplinary psychoeducational intervention, unfolds over five instructive sessions, each addressing key aspects of hygiene and COVID-19 awareness (Supplementary Material) (16). These sessions cover topics such as hand hygiene, facial contact avoidance, sneezing etiquette, mask-wearing, and social distancing. To make the learning experience engaging and child-friendly, we utilize specialized exercises and games. For ease of accessibility, the full English translation of our CARTOON program is provided in the Supplementary Material.

Our program introduces the “CCP heroes” (Supplementary Material). These heroes, namely Captain Soapy Hands, Mister Sneeze, Do not Touch Tina, Flying Mask-up Mona, and Hero of the Distance, are depicted in a comic style designed by Dr. Philipp Steinbauer at the Comprehensive Center for Pediatrics (CCP) of the Medical University of Vienna. The CCP heroes effectively convey fundamental rules to prevent the spread of SARS-CoV-2. Posters featuring these self-designed heroes were prominently displayed in the kindergarten environment, serving as regular reminders for children to adhere to hygiene measures.

Moreover, our psychoeducational program spanned five days, with each daily session lasting 60 min. Conducted in groups of up to six children, the sessions were facilitated by trained staff members, ensuring an interactive and informative learning experience. To minimize response bias, interviewers were trained to use neutral phrasing, avoid leading questions, and ensure a comfortable, non-judgmental environment. Visual aids (such as illustrations) were incorporated to support understanding. Additionally, children were encouraged to provide their own answers without external influence from peers or caregivers. Participant adherence to the program was monitored throughout the intervention. Attendance records were maintained for each session, and completion rates were assessed at the end of the program. There were no additional drop-out rates during the study period.

### 2.3 Questionnaires

#### 2.3.1 Socioeconomic questionnaire

All parents of the participants completed a questionnaire collecting socioeconomic data including age, profession, and family living situation. The questionnaire was completed at the start of the study before the intervention phase (Figure 1).

#### 2.3.2 COVID-19 quiz

The quiz consisted of a 12-item questionnaire and covers theoretical and practical knowledge about viral infections and hygiene measures. Content validity of the knowledge test was reviewed by an expert panel consisting of three psychologists with expertise in children health, one kindergarten teacher, and one pediatrician.

The quiz was completed by children, parents, and teachers at baseline, immediately after, and three months after the intervention phase by both the control group and intervention group participants. In addition, all included children and teachers completed the quiz three months after the intervention phase (Figure 1). While one point was awarded for each correct answer, no points were awarded for each incorrect answer.

Since children of this age lack the ability to complete a questionnaire independently, a psychologist conducted interviews with the children and filled out the questionnaire on their behalf. An overview of a translated version into English of the COVID-19 quiz is provided in the Supplementary Material.

##### 2.3.2.1 Emotional scale

Since it is often hard for children to describe their own feelings with words, we used three of the emotional scales of Gräßler et al. (17). These scales use child-friendly illustrated pictures and can be used to support the children in naming their feelings including emotional wellbeing, fear, and burden.

### 2.4 Outcomes

#### 2.4.1 Primary outcome

The primary outcome was the change in the children's emotional state and knowledge before and after participation in the educational program. To evaluate the emotional and knowledge outcome of children we employed two assessment tools. For the assessment of emotional wellbeing, fear, and burden during the pandemic we used an emotional scale which measures anxiety. Furthermore, we investigated the impact of CARTOON on the participants' theoretical understanding and hygiene practices related to COVID-19 with a quiz.

#### 2.4.2 Secondary outcome

Our secondary outcome was the long-term impact of CARTOON three months after the educational program. We investigated if the psychoeducational intervention impacts emotional and knowledge outcome, as well as retention of acquired theoretical and practical knowledge three months after the intervention. In addition, we investigated which covariates influence the knowledge regarding COVID-19 and hygiene measures.

### 2.5 Statistical analysis

Descriptive statistics were used to summarize baseline characteristics of recruited participants. In order to test differences between the two groups with regard to theoretical and practical knowledge, as well as emotion *t*-tests for independent samples were performed.

Categorical variables are in absolute frequencies and percentages. Continuous variables are listed in means and standard deviations and were presented in tables. For the analysis of covariates to predict knowledge regarding COVD-19 and hygiene measures a linear regression model was performed. The following covariates were included: age, gender, education of mother, number of children in the household, cramped living conditions, and mental stress in the family. Statistical analysis was performed using SPSS 24 (IBM Corporation, Armonk, NY, USA). A *p*-value of < 0.05 was considered as statistically significant.

#### 2.5.1 Sample size calculation

Sample size estimation was based on a previously published meta-analysis by Taylor et al. examining the relationship between science education interventions and effect sizes (18). According to this meta-analysis we assumed a minimum effect size of 0.37. Based on an alpha of.05, sample size estimates indicated that we would be able to detect a difference with a sample size of 52, based on an effect size of 0.37 at a power of. 80. However, as no previous research was available on which to accurately base these effect sizes, these estimates were considered as vague approximations.

## 3 Results

Of the 57 children initially enrolled in the study (26 intervention, 31 control), complete assessment data were only available for 53 participants. Four children from the control group were excluded from the final analysis due to entirely missing assessment data. Thus, the final analyzed sample consisted of 26 children in the intervention group and 27 children in the control group. Therefore 53 children finished the CARTOON educational program between January and May 2021. Table 1 shows the population characteristics of study participants, while Table 2 provides the socioeconomic characteristics of the parents including age, education, language, and living conditions. There were no significant differences between the intervention and control groups regarding population and socioeconomic characteristics.

**Table 1:** Baseline characteristics.

Characteristic	Control group (*n* = 27)	Intervention group (*n* = 26)	*p*-value
Children characteristics			
Female gender, (*n* %)	13 (48)	10 (38)	.412
Male gender, (*n* %)	14 (52)	16 (62)	.412
Age, mean	4.5 ± 0.99	5.0 ± 0.90	.152
Age distribution, (*n* %)			
3–4 years	13 (48)	13 (50)	
5–6 years	14 (52)	13 (50)	

Counts and percentages are presented as *n* (%) and parameters are expressed as mean ± SD. Percentages are based on the respective group. To calculate the *p*-value a *t*-test was used for parametric continuous variables and a Mann–Whitney-*U* test was used for non-parametric continuous variables.

**Table 2:** Socioeconomic characteristics.

Socioeconomic characteristics	Control group (*n* = 27)		Intervention group (*n* = 26)		*p*-value	
	Mother	Father	Mother	Father	Mother CG vs. IG	Father CG vs. IG
Parental Age, years, mean ± SD	37.1 ± 4.5	40.2 ± 5.3	36.9 ± 6.4	39.9 ± 7.4	.860	.866
Nationality, (*n* %)						
Austria	23 (86)	19 (70)	22 (84)	22 (84)	.870	.138
Serbia/Bosnia/Croatia	2 (7)	2 (7)	2 (8)	2 (8)		
Others	2 (7)	6 (23)	2 (8)	2 (8)		
Spoken language, (*n* %)					.541	.321
German	18 (66)	17 (63)	17 (65)	19 (73)		
Others	2 (8)	2 (8)	0 (0)	0 (0)		
German/Serbian/Croatian	1 (4)	1 (4)	1 (4)	2 (8)		
German/Others	6 (22)	7 (25)	8 (31)	5 (19)		
Marital status. (*n*%)					.125	.184
Single	1 (4)	1 (4)	4 (15)	4 (15)		
Cohabitation	8 (30)	8 (30)	3 (12)	3 (12)		
Married	15 (54)	15 (54)	19 (73)	19 (73)		
Separated	1 (4)	1 (4)	0 (0)	0 (0)		
Widowed	0 (0)	0 (0)	0 (0)	0 (0)		
Divorced	2 (8)	2 (8)	0 (0)	0 (0)		
Education, (*n* %)					.230	.841
Elementary school	0 (0)	0 (0)	0 (0)	0 (0)		
General secondary school/Prevocational year	1 (4)	2 (8)	0 (0)	2 (8)		
Final apprenticeship exam	3 (11)	6 (22)	5 (19)	7 (27)		
Secondary vocational school	1 (4)	0 (0)	2 (8)	1 (4)		
Grammar school/Secondary vocational college	4 (16)	4 (16)	8 (31)	3 (11)		
University	18 (66)	15 (54)	11 (38)	13 (50)		
Siblings, Yes, (*n*%)	18 (67%)		15 (58%)		.490	
Firstborn, Yes, (*n*%, Percentage related to siblings)	8 (44)		7 (47)		.982	
Living Conditions, (*n*%)					.192	
Flat	25 (92)		21 (81)			
House	2 (8)		5 (19)			
Place, (*n* %)					.364	
City	25 (92)		21 (81)			
Country	2 (8)		5 (19)			
People in household, mean ± SD	3.6 ± 0.9		3.6 ± 1.0		1.0	
Quality of living, (*n* %)					.267	
Good	22 (81)		23 (88)			
Sufficient	5 (19)		3 (12)			
Poor	0 (0)		0 (0)			
Financial conditions, (*n* %)					.289	
Barely	3 (10)		3 (11)			
Sufficient	16 (60)		19 (74)			
Prosperous	8 (30)		4 (15)			
Support for childcare, (*n* %)					.085	
No	16 (60)		20 (77)			
Yes	11 (40)		6 (23)			
Family burden, (*n* %)					.594	
No	24 (89)		21 (81)			
Yes	3 (11)		5 (19)			

Counts and percentages are presented as *n* (%) and parameters are expressed as mean ± SD. Percentages are based on the respective group. To calculate the *p*-value a *t*-test was used for parametric continuous variables and a Mann–Whitney-*U* test was used for non-parametric continuous variables.

### 3.1 Baseline assessment

Fifty-three children completed the baseline assessment including the corona quiz and the assessment of anxiety (Table 3). There were no significant differences between the control and intervention group when comparing the means of theoretical knowledge about COVID-19 (4.1 ± 2.3 vs. 3.9 ± 2.4; *p* = .76) and hygiene knowledge (6.3 ± 2.3 vs. 5.9 ± 2.3; *p* = .53) at baseline. Moreover, we did not find a difference between the control and intervention group regarding overall anxiety (1.3 ± 2.9 vs. 1.1 ± 1.7; *p* = .77) and anxiety regarding COVID-19 (5.1 ± 3.8 vs. 5.6 ± 2.5; *p* = .57) at baseline. However, when comparing overall anxiety with anxiety regarding COVID-19 within the groups there was a statistically significant difference in both groups (control group: 1.3 ± 2.9 vs. 5.1 ± 3.8, *p* = .01; intervention group: 1.1 ± 1.7 vs. 5.6 ± 2.5, *p* < .01) (Table 3).

**Table 3:** Knowledge outcomes.

	Control Group (*n* = 27)		*p*-value (baseline vs. short term)	Intervention Group (*n* = 26)		*p*-value (baseline vs. short/long term)	*p*-value (control BL vs. intervention BL)
Knowledge about COVID-19 in general (8 Questions: 1–5, 16–18), mean ±SD	Baseline	Short Term		Baseline	Short Term		
	4.1 ± 2.3	4.0 ± 1.9	.86	3.9 ± 2.4	4.6 ± 1.9	.27	.76
	Baseline	Long Term		Baseline	Long Term	.	
	4.1 ± 2.3	4.4 ± 2.0	.60	3.9 ± 2.4	5.9 ± 2.0	.003*	.76
Knowledge about hygiene measures (10 Questions: 6–15), mean ±SD	Baseline	Short Term		Baseline	Short Term		
	6.3 ± 2.3	6.2 ± 2.0	.86	5.9 ± 2.3	6.3 ± 2.6	.58	.53
	Baseline	Long Term		Baseline	Long Term		
	6.3 ± 2.3	6.7 ± 2.3	.51	5.9 ± 2.3	8.2 ± 2.2	<.001*	.53
Anxiety now Median, mean ± SD	Baseline			Baseline			
	0, 1.3 ± 2.9			0, 1.1 ± 1.7			.77
Anxiety COVID-19 Mean ± SD	Baseline	Short Term		Baseline	Short Term		
	5.1 ± 3.8	4.9 ± 3.1	.39	5.6 ± 2.5	2.2 ± 1.7	.01*	.58

* Statistically significant (*p* < 0.05); control group *n* = 27, intervention group *n* = 26.

Parameters are shown as mean ± SD or Median. To calculate the *p*-value a t-test was used for parametric continuous variables and a Mann–Whitney-U test and Wilcoxon signed-rank test was used for non-parametric continuous variables.

BL, baseline; ST, short term; LT, long term.

### 3.2 Short-term assessment (immediately after CARTOON)

Fifty-three children completed the short-term assessment (Table 3). There were no significant differences when comparing the baseline knowledge about COVID-19 with the short-term knowledge about COVID-19 within the control group (4.1 ± 2.3 vs. 4.0 ± 1.9; *p* = .86) as well as the intervention group (3.9 ± 2.4 vs. 4.6 ± 1.9; *p* = .27). When comparing the baseline knowledge about hygiene measures with the short-term knowledge about hygiene measures, no significant differences within the control (6.3 ± 2.3 vs. 6.2 ± 2.0; *p* = .86) and intervention group (5.9 ± 2.3 vs. 6.3 ± 2.6; *p* = .58) were found.

Participants in the intervention group exhibited significantly lower anxiety scores after the short-term assessment when compared with the baseline assessment (2.2 ± 1.7 vs. 5.6 ± 2.5; *p* = .01). However, there was no significant difference within the control group when comparing the baseline scores of COVID-19 related anxiety with short-term scores (5.1 ± 3.8 vs. 4.9 ± 3.1; *p* = .39) (Table 3).

### 3.3 Long-term assessment (3 months after CARTOON)

Fifty-three children completed the long-term assessment (Table 3). Control group children showed no significant differences in knowledge about COVID-19 when comparing the baseline with the long-term assessment scores (4.1 ± 2.3 vs. 4.4 ± 2.0, *p* = .60). However, children assigned to the intervention group showed an increase in knowledge about COVID-19 when comparing baseline and short term scores (3.9 ± 2.4 vs. 5.9 ± 2.0, *p* = .003).

There were no significant differences in knowledge about hygiene measures when comparing the baseline (6.3 ± 2.3) with the long-term assessment scores (6.7 ± 2.3; *p* = .51). However, children included in the intervention group showed significantly better knowledge about hygiene measures in the long-term assessment when compared to the baseline assessment scores (7.2 ± 2.2 vs. 5.9 ± 2.3, <0.001).

### 3.4 Regression analysis

Results of multivariable linear regression analysis of risk factors to predict knowledge scores about COVID-19 and hygiene measures are provided in Table 4. Lower age of the children and lower education of the mother were both significantly predictive for lower knowledge regarding COVID-19 and hygiene measures in children. In addition, mental stress in the family was also associated with lower knowledge regarding COVID-19 and hygiene measures (Table 4).

**Table 4:** Linear regression model.

	Estimate	*p*-value
Knowledge COVID-19		
Age	0.183	<.01*
Gender	−0.057	.42
Education of mother (university)	0.199	<.01*
At least 2 children in the household	−0.015	.76
Cramped living conditions	−0.057	.56
Mental stress in the family	0.071	.04*
Knowledge hygiene measures		
Age	0.116	<.01*
Gender	−0.028	.64
Education of mother (university)	0.066	<.01*
At least 2 children in the household	0.015	.73
Cramped living conditions	−0.077	.37
Mental stress in the family	0.102	.03*

* Statistically significant (*p* < 0.05).

A stepwise selection of risk factors was performed;—indicates not an important risk factor with additional value for prediction in this model.

R-square knowledge COVID-19: 0.46; R-square knowledge hygiene measures: 0.35; control group *n* = 27, intervention group *n* = 26.

## 4 Discussion

The COVID-19 pandemic had profound effects on both physical and mental health, particularly among children. The prolonged disruption of daily routines, school closings, social isolation, and increased stress levels have contributed to a rise in mental health challenges among children (19). Studies have shown increased rates of anxiety, depression, post-traumatic stress symptoms, and behavioral difficulties in children and adolescents during the pandemic (6, 20–22).

In this study, we introduced a psychoeducational program in kindergartens, to develop effective educational strategies for preparing children, parents, and teachers to navigate the challenges presented by the ongoing pandemic. The educational program contributed to a significant knowledge increase about COVID-19 and hygiene measures three months after the intervention. Moreover, the intervention leads to a significant reduction in anxiety scores related to COVID-19. In our regression model, lower age of children, lower education of the mother and presence of mental stress in the family were significantly predictive of lower knowledge regarding COVID-19 and hygiene measures. To our knowledge, this is the first randomized controlled trial, investigating the impact of an educational program on knowledge, hygiene practices, and anxiety levels regarding the COVID-19 pandemic among kindergarten-aged children.

Schools and kindergartens play a crucial role in providing a structured environment for children to learn and develop social skills such as self-confidence, friendship, empathy, respect, and responsibility. This enables them to become active members of a community based on solidarity. Paakkari and Okan emphasized the importance of enhancing health literacy within educational settings (23). After the pandemic we have to rethink the role of the kindergarten and school. Teachers should proactively promote health among children from an early age by encouraging healthy habits like personal hygiene (24).

When targeting kindergarten children and school children with health messages, it is crucial to incorporate positive, engaging, entertaining, fun, and humorous elements while ensuring age-appropriate accuracy. Cartoons, popularized by Disney have a long history promoting learning and interaction in children (25). In particular, cartoons have demonstrated significant value, as they can effectively reinforce desired behaviors through direct observation, a critical aspect of behavioral learning (26–28).

To address the urgent need for targeted COVID-19 prevention messages for kindergarten children, it is logical to employ an entertainment education approach based on cartoons. With our CARTOON, we provide a successful free education intervention-package consisting of five heroes including exercises about hygiene measures and knowledge about COVID-19. The full program is included for download in the Supplementary Material.

### 4.1 Impact of CARTOON on knowledge

To the best of our knowledge, there is currently only one cartoon-based educational program on hygiene measures for children. However, it is important to note that that program, named “The Magic Glasses,” does not function as an educational intervention against COVID-19. Instead, it serves as a hygiene education intervention package specifically designed to prevent worm infections in Chinese schoolchildren (28). In contrast, the CARTOON program effectively conveys fundamental rules to prevent the spread of SARS-CoV-2, and could be easily adapted to other situations associated with the spread of respiratory pathogens, showing propensity to generalizability. CARTOON has demonstrated a significant enhancement in knowledge about COVID-19 and adherence to preventive measures three months post-intervention. This suggests its potential as a valuable tool for future pandemics. By employing child-friendly teaching methods and engaging visuals, we effectively communicated information to preschool children, facilitating a deeper understanding of knowledge and preventive measures. Despite the notable impact of our educational program on knowledge, the regression model highlights two key factors that warrant further consideration. Specifically, the age of the participant and maternal education emerged as particularly influential in the process of knowledge acquisition. In fact, children's ability to form a real representation of important life events develops gradually. Around the ages 3–5, children start to understand basic concepts, but a more nuanced understanding typically develops between the ages 7–10 (29, 30). As children grow and their cognitive abilities mature, they progress from concrete thinking, which is focused on tangible, observable things, to abstract thinking, which involves understanding symbols, metaphors, as well as hypothetical, and more complex situations. Abstract thinking strongly relates to the maturation of cognitive abilities, the latter particularly mediated by surrounding factors such as maternal education (30). Maternal education, in particular, has been found to have a significant impact on various aspects of children's knowledge acquisition and overall development, including: cognitive development, language development, educational attainment, social and emotional behavior, and nutrition (31–33).

### 4.2 Impact of CARTOON on anxiety

Usually, knowledge can have a significant impact on psychic well-being in various ways. When individuals have clear, reliable knowledge about a situation, they are better equipped to understand potential risks and develop effective coping strategies. Ambiguity and uncertainty, on the other hand, can contribute to heightened anxiety. The same is true in the context of health care and crisis situations (34–36).

The COVID-19 pandemic has not only posed significant physical health risks but has also had a substantial impact on the mental well-being of individuals, including children.

Importantly, our CARTOON intervention not only focused on knowledge acquisition but also addressed children's emotional well-being. Participants in the intervention group experienced a significant reduction in anxiety scores related to COVID-19, whereas the control group did not show significant changes. This finding suggests that CARTOON not only provided children with knowledge but also helped alleviate their fears and anxiety surrounding the pandemic. Interestingly, higher levels of COVID-19-related knowledge and knowledge about preventive measures were associated with reduced emotional and behavioral problems in children in a study by Wang and colleagues (37). This is also in line with a study by Zhou and colleagues who showed a high prevalence of psychological health problems among adolescents, which were negatively associated with the level of knowledge about COVID-19 (5). By using child-friendly illustrated pictures and engaging teaching sessions, we created an environment where children felt empowered and equipped to cope with the challenges posed by the virus.

### 4.3 Limitations

Our study has several limitations. First, our sample size was relatively small, which may limit the generalizability of the findings. Although the results demonstrate significant improvements in hygiene knowledge and anxiety reduction, larger-scale studies are required to validate these effects in broader populations. Additionally, the study only included a three-month follow-up, making it difficult to assess the long-term retention of knowledge and behavior change.

Second, the study was conducted in two kindergartens in Vienna, Austria. The specific cultural and educational context may influence the results, and the findings might differ in other settings or different countries with variations in educational systems, and cultural differences in health education approaches. Third, the emotional scales and quizzes, while adapted for children, may still have limitations in accurately capturing the full range of emotions and knowledge in young children. However, we have used only validated tools in order to minimize this bias.

Another potential limitation of this study is the possibility of response bias in children's interviews. Due to their developmental stage, young children may interpret questions differently, provide socially desirable answers, or be influenced by the interviewer's phrasing or demeanor. While efforts were made to minimize these biases through structured interview techniques, neutral questioning, and the use of visual aids, the possibility of misinterpretation or response distortion cannot be entirely ruled out.

In addition, other factors, such as prior exposure to hygiene education or parental attitudes toward hygiene, may also play a role in shaping children's knowledge and responses to the intervention. Since these data were not collected in this study, their potential influence on the results remains unknown.

## 5 Conclusions

The results of our study have important implications, revealing that a cost-effective and easily accessible brief intervention can effectively educate and empower the public during and beyond the COVID-19 pandemic. We promote the importance of health education and primary prevention of infectious diseases in kindergarten settings. Our goal as to raise awareness about the significance of these measures and their continued relevance in a post-pandemic context. By instilling good hygiene practices early on, the CARTOON program seeks to establish a foundation that contributes to overall health and well-being of preschool children, offering lasting benefits beyond the challenges posed by the COVID-19 pandemic.

While our findings suggest that a cartoon-based intervention is effective in improving hygiene knowledge and reducing anxiety in preschool children, further research is necessary to assess its applicability in diverse cultural and economic contexts. Future studies should explore feasibility, adaptability, and scalability before large-scale implementation is considered.

Public health authorities should consider incorporating an educational intervention, similar to the one employed in our study, as a means to empower communities and foster significant changes in behavior.

## Data Availability

The raw data supporting the conclusions of this article will be made available by the authors, without undue reservation.
