# Supplementary material for: CARTOON-based educational intervention for children to foster hygiene knowledge and emotional resilience in preschool children: a randomized controlled trial
Source: Front Pediatr. 2025 Apr 11;13:1514793. doi: 10.3389/fped.2025.1514793 (PMC12021609; doi:10.3389/fped.2025.1514793)
Supplement: Supplementary file 1 [file Datasheet1.pdf]

SUPPLEMENTARY MATERIAL

1. PSYCHOEDUCATIONAL PROGRAM 2

- A. INTRODUCTION: 2
- B. THEORETICAL BACKGROUND: 3
- C. TARGET GROUP: 4
- D. OBJECTIVE: 4
- E. STRUCTURE: 4
- F. IMPLEMENTATION OF THE MODULES: 5

2. THE CCP-HEROES 8

- A. BLUE HERO 8
- B. GREEN HERO 9
- C. ORANGE HERO 10
- D. RED HERO 11
- E. PINK HERO 12

3. CORONA QUIZ 13

- A. QUESTION 1 13
- B. QUESTION 2 14
- C. QUESTION 3 15
- D. QUESTION 4 16
- E. QUESTION 5 17
- F. QUESTION 6 18
- G. QUESTION 7 19
- H. QUESTION 8 20
- I. QUESTION 9 21
- J. QUESTION 10 22
- K. QUESTION 11 23
- L. QUESTION 12 24
- M. QUESTION 13 25
- N. QUESTION 14 26
- O. QUESTION 15 27
- P. QUESTION 16 28
- Q. QUESTION 17 29
- R. QUESTION 18 30

1. Psychoeducational Program

„With Superheroes against the Coronavirus!“

A psychoeducational intervention program to promote knowledge and hygiene measures and dispel fear during the COVID-19 pandemic for children between the age of 3 and 15 years.

a. Introduction:

The psychosomatic outpatient day clinic at the Department of Pediatrics at the Medical University of Vienna children and adolescents between the ages of 3 and 15 are cared for in an interdisciplinary team according to a multimodal treatment concept. It consists of a kindergarten group for children between 3 and 6 years as well as two school classes for school-aged children and adolescents.

As a required measure to contain COVID-19 the day clinic had to be closed between March 16 and May 18, 2020. The reopening of the clinic was only possible by complying to highly restrictive hygiene measures (Berger & Kurz, 2020). These include, on the one hand, general measures (wearing a face mask, handwashing, not touching the face, sneeze-cough-etiquette, physical distancing) and on the other hand, regular nasopharyngeal swabbing for COVID-PCR-testing of all patients and members of staff.

The universal threat posed by the virus, the required measures and regular medical examinations can lead to an increased experience of stress in children and adolescents and, subsequently, feelings of anxiety, depression, and uncertainty („6 ways parents can support their kids through the corona virus disease (COVID-19) outbreak “, 2020). To minimize negative effects of this stressful situation, professionals with psychological, educational, medical, and artistic background collaborated to develop an interdisciplinary training concept for COVID-prevention aimed at children and adolescents. For this purpose, training materials, including games, exercises, and information, were created and then adapted for different age groups. They can be used for educational purposes at the Department of Pediatrics, but also in other medical and therapeutic facilities (doctor’s offices, outpatient clinics) as well as kindergartens, schools, and after-school care centers.

Our interdisciplinary training concept is intended to have an impact on kindergarten pedagogy during the pandemic and provide conditions under which kindergartens can reopen and remain open. We consider our program to be an important contribution to increasing compliance and adherence to hygiene measures and reducing fears of children and their caregivers.

b. Theoretical background:

Medical interventions can cause psychological and/or physiological stress in children due to the associated perception of danger, anticipation of pain and experienced loss of control (Kain, Mayes, Caldwell-Andrews, Karas, McClain, 2006). As a result, the compliance, therefore the willingness and motivation of patients to follow a medical regimen and participate in medical procedures, declines. In the long-term, increased stress levels can jeopardize mental and physical health (Brewer, Gleditsch, Syblik, Tietjens & Vacik, 2006). At the psychological level, it may lead to anxiety and prevent the development of appropriate coping skills. To reduce patients' emotional distress and improve compliance, psychoeducational patient trainings have already been developed for many chronic diseases (Parfy, Lenz, & Schuh, 2016).

According to Barlow and Ellard (2004), many of these psychoeducational interventions are based on theoretical concepts such as self-efficacy and empowerment. Empowerment means having control over one's life and adopting a proactive attitude. This can have a positive impact on motivation, thought patterns, and emotional responses. Similarly, education and knowledge about a disease and positive assumptions about the effects of treatment have been shown to strengthen compliance (Laux & Dietmaier, 2009).

Psychoeducational training programs usually include various modules to impart knowledge about one's own body, disease, and treatment and address preventive and maintaining factors regarding the disease. They are becoming increasingly important in the field of pediatrics as a tool for adequate management of physical illness (Barlow & Ellard, 2004). Studies have demonstrated that stress and anxiety can be reduced through targeted information and by learning appropriate coping strategies (Brewer et al., 2006). According to Barlow and Ellard (2004), knowledge about health is best transferred through highly interactive and individualized information in children. For example, therapeutic play in preparation for medical interventions can reduce anxiety and shows positive effects on pain management (Da Silva et al., 2017). Providing children with specific information about a medical procedure and its purpose can counteract unrealistic expectations and fantasies about the intervention.

The foundation for patient education is a sufficient body concept, i.e., knowledge about one’s own body and its function. Body concepts vary greatly in differentiation depending on the developmental age of the child. Preschool children for example demonstrate very vague ideas about their body, can name only 2 to 6 internal organs, and have rudimentary knowledge about their functions (Lohaus & Ball, 2006). Therefore, the primary need in patient education in pediatrics is to provide children with body knowledge as a foundation for further understanding of disease and treatment. Fernandes et al. (2014) hypothesize that child-friendly preparation before a medical intervention supports the development and use of favorable coping skills. Children have the ability to cope with stressful situations and learn goal-oriented coping behaviors. In young children, coping skills are initially limited, but by adolescence a broad spectrum of coping skills has developed, which can also be used in a more flexible way (Lohaus & Ball, 2006). Within the framework of training programs, the development of coping skills can be promoted specifically depending on age group and personal resources.

c. Target group:

Children and adolescents with complex psychological and psychosomatic disorders are treated at the psychosomatic outpatient day clinic. These include somatoform or functional disorders (e.g. functional abdominal pain), elimination disorders (enuresis, encopresis), feeding disorders as well as attachment and interaction disorders. In addition, children with psychological and behavioral factors associated with disorders classified elsewhere (including affective disorders, anxiety disorders, hyperkinetic disorders), as well as children with chronic illnesses, in which psychological and psychosocial stress play an important role, are treated. These patients are at increased risk for a severe course of disease if infected with the corona virus, which is why it is crucial that all protective measures are adhered to. The training program is intended for patients of all age groups, with individual modules for kindergarten or school age. In addition to the application at the outpatient day clinic, the training program can be used in the entire children's hospital, as well as other suitable facilities or even in families. This way as many children and adolescents as possible will be able to benefit from this project.

d. Objective:

We aim to achieve several objectives with the implementation of our COVID-prevention program. The first goal is to transfer knowledge and competence regarding the COVID-19-disease and necessary protective measures age-appropriately. The cognitive and emotional stage of development of children should be considered when imparting knowledge. Therefore, we aim to increase motivation of participants through experiential learning. We intend to strengthen children’s and adolescent’s sense of competence and self-efficacy and as a result reduce feelings of helplessness and anxiety. In addition, our project aims to improve children's compliance during the nasopharyngeal swab test and their cooperation in implementing the necessary protective measures.

e. Structure:

Six different modules were developed for the implementation of this project, in which different focal points of knowledge transfer were addressed. Modules 1 - 4 deal with the implementation of the five most important protective measures: Hand hygiene, not touching the face, cough-sneeze-etiquette, wearing a face mask/shield and keeping distance. To increase children's motivation to participate in the teaching program and to explain the five protective measures in child-friendly terms, we used the "CCP Superheroes" (Mister Sneeze, Captain Soapy Hands, Do not Touch Tina, Flying Masked Mona and Hero of the Distance), developed by Dr. Steinbauer for the Comprehensive Center for Pediatrics (CCP). Posters of the superheroes can be viewed at multiple locations at the outpatient day clinic, as well as throughout the pediatric clinic, and are used to continually remind patients to comply with the measures. These can also serve as conversation starters to discuss the five safety measures with children (What rules does the child recognize from the poster? What are the names of the superheroes? What are their superpowers? Which superhero does the child like best and why?). Module 5 is designed to educate and prepare children for the

regular nasopharyngeal swab (or throat swab for young children). By preparing children for this, often perceived as invasive, medical examination, potential fear and uncertainty can be reduced and compliance can be strengthened.
Finally, general knowledge about the Corona virus is transferred in Module 6. Through information and education, patients will feel more in control during the current situation around COVID-19 and that they can make a positive contribution.
In order to provide children and adolescents with the best possible experience in experiential learning, suggestions for psychologists, educators, and parents were created in the form of index cards. These index cards contain a collection of ideas for each module (see also: Implementing the Modules). Furthermore, the children can collect stamps for each completed module in a passport (Superhero Passport).

f. Implementation of the modules:

(1) Hand hygiene & not touching faces:

The goal is to teach children the necessity and proper implementation of hand hygiene and not touching their faces with their hands in this module. Experiments are a good way for preschool and school-age children to understand the need for proper hand hygiene. For example, by putting glitter on their hands, children can experience how quickly bacteria and viruses found on their hands can spread around the room and on the body.
Another helpful experiment suitable for teaching preschool and school-age children how to implement proper hand hygiene is painting the hands with acrylic paint. The children are then instructed to try to wash the paint off first without soap, which is difficult and then with soap, which leads to sucess. This way, children can playfully experience the importance of thorough hand washing with soap. To facilitate and remind them that hand washing needs to be done for at least 30 seconds, poems or songs that revolve around the topic of hand hygiene can be sung together or hung up next to the sink. An example of a poem that fits this theme is shown in Figure 1 and can be found in the appendix. To make hand washing even more playful, soap making is another great option. Instructions can be found at the following link:
https://praxistipps.focus.de/seife-mit-kindern-selber-machen-eine-bastelanleitung_99487

(2) Cough-sneeze etiquette:

In this module children learn about the necessity of sneezing into the crook of the arm. For this purpose, a video can be used, which explains what happens when we sneeze, how viruses spread and why sneezing into the crook of the arm helps to prevent this. The video is suitable for younger as well as older children. https://www.youtube.com/watch?v=dmql3J1CCsM&feature=emb_title
Especially for younger children, the use of mnemonic phrases (possibly in the form of a nsuperhero saying) can also make it easier for them to remember this rule better.

(3) Wear mask/shield:

The goal of this module is to help children understand the necessity of wearing a mask or shield and to develop strategies for making this as comfortable as possible. For this purpose, a cotton

mask can be sewn or purchased and then decorated together, for example, using fabric paints.
If children find wearing the mask particularly uncomfortable, they can work with distraction strategies (5-4-3-2-1 exercise; finding red/ blue/ yellow/ etc. 5 times in their environment as quickly as possible; counting) and affirmations ("I am a superhero/ superheroine and protect myself and others with the mask").

(4) Distance

In this module children are introduced to the unit of measurement for one or two meters. To give preschool and school-age children a sense of how long one or two meters are, materials such as a string or duct tape can be used. In addition, figures made of cardboard with a width of one meter, such as a superhero or a baby elephant, can be made together to make the measurement more tangible and to create an image that makes it easier to remember it in everyday life.
At kindergarten age, physical closeness, especially to caregivers and other children, cannot and should not be prohibited. The "distance" module is therefore intended to help children assess certain risks in times of pandemic better and to strengthen compliance with hygiene measures.

(5) Nasopharyngeal swab

The aim of this module is to prepare the children for nasopharyngeal swabbing (or throat swabbing for young children) for Corona testing. For this purpose, we attempted to “transform” the swab into a search device (reframing), called SUSU (“Super-Suchgerät”; german for “super search device”). A drawing of SUSU can be found in the appendix. Child-friendly mnemonic sentences such as „Corona versteckt? SUSU entdeckt!“ ("Corona hidden? SUSU detects!") und „Mund auf, Zunge `raus, SUSU spürt die Viren auf! Du bist ein Superhero!“ ("Mouth open, tongue out, SUSU tracks down the viruses! You are a superhero!") ought to make the procedure more tolerable to children. For this purpose, dolls with a large nose or mouth opening can also be used for the children to practice the procedure under guidance. In addition, identification with the cartoon characters (superheroes) can increase motivation and cooperation and reduce possible fears. Through their compliance children can “support” the superheroes and their work, which strengthens self-efficacy. Even so distraction strategies are especially important during the nose swab and should be worked on with the child. Some ideas regarding this are:
- Buzzing like a bee - this automatically makes the child's breathing more even and allows them to focus their attention on the buzzing
- Thinking of a corresponding animal for each letter in the alphabet, starting from A.
- Distraction by other senses (what do I see, what do I feel, what do I smell; 5-4-3-2-1 exercise; finding red/ blue/ yellow/ etc. 5 times in their environment as quickly as possible)

(6) General knowledge

In this module children are provided with information on the topic of corona in a way, that is suitable for their age and by using simple language. The aim is for them to gain a basic understanding of the topic and the social implications, such as: *What is a virus? What can happen if you carry the virus inside you? How can our body protect itself from the virus (immune system)?*

How does infection occur? Where does the Corona virus come from and why is it called that? Is there already a medication or a vaccine? and so on. Furthermore, changes brought about by the pandemic, such as closing schools, requiring masks in public places, not being able to visit grandparent, etc. are discussed. This module is designed for children aged 6 years or older. Supporting materials can also be used, such as:

- https://www.mindheart.co/descargables (materials available in different languages)
- https://www.daniela-lempertz.de/wp-content/uploads/2020/03/Mission-Zuhause-Bleiben-2020.pdf
- George and the Corona- Virus coloring book (see appendix)
- https://www.gemeinsamlesen.at/fileadmin/corona/Corona-Schueler-ab-3.-Klasse.pdf
- https://www.gemeinsamlesen.at/fileadmin/corona/Corona-Schueler-ab-9.-Schulstufe.pdf
- Brochure: Tips for families on dealing with the Corona Crisis: http://intranet.akhwien.at/default.aspx?did=145991

Kindergarten age: At the beginning of the program, children receive a training passport (see Figure 2 and Appendix), which contains stamps for modules 1 to 5. When all five stamps have been collected in the passport, children are issued a superhero certificate signed by their personal favorite superhero (see Figure 4).

School age: The materials of the 6 different modules are worked through with children from around the age of 6. Once they have gone through the training phase, they are asked to complete a quiz ("Are you a Corona Expert?") (see Figure 3 and Appendix). Upon successful completion of the modules and the quiz, the children are issued a superhero certificate signed by their personal favorite superhero (see Figure 4).

All training materials are collected and available in a folder. The certificate can be created and customized at: https://www.onlinewahn.de/generator/urkunde.php. An example of a self-created certificate can be seen in Figure 4.

2. The CCP-Heroes

a. Blue Hero

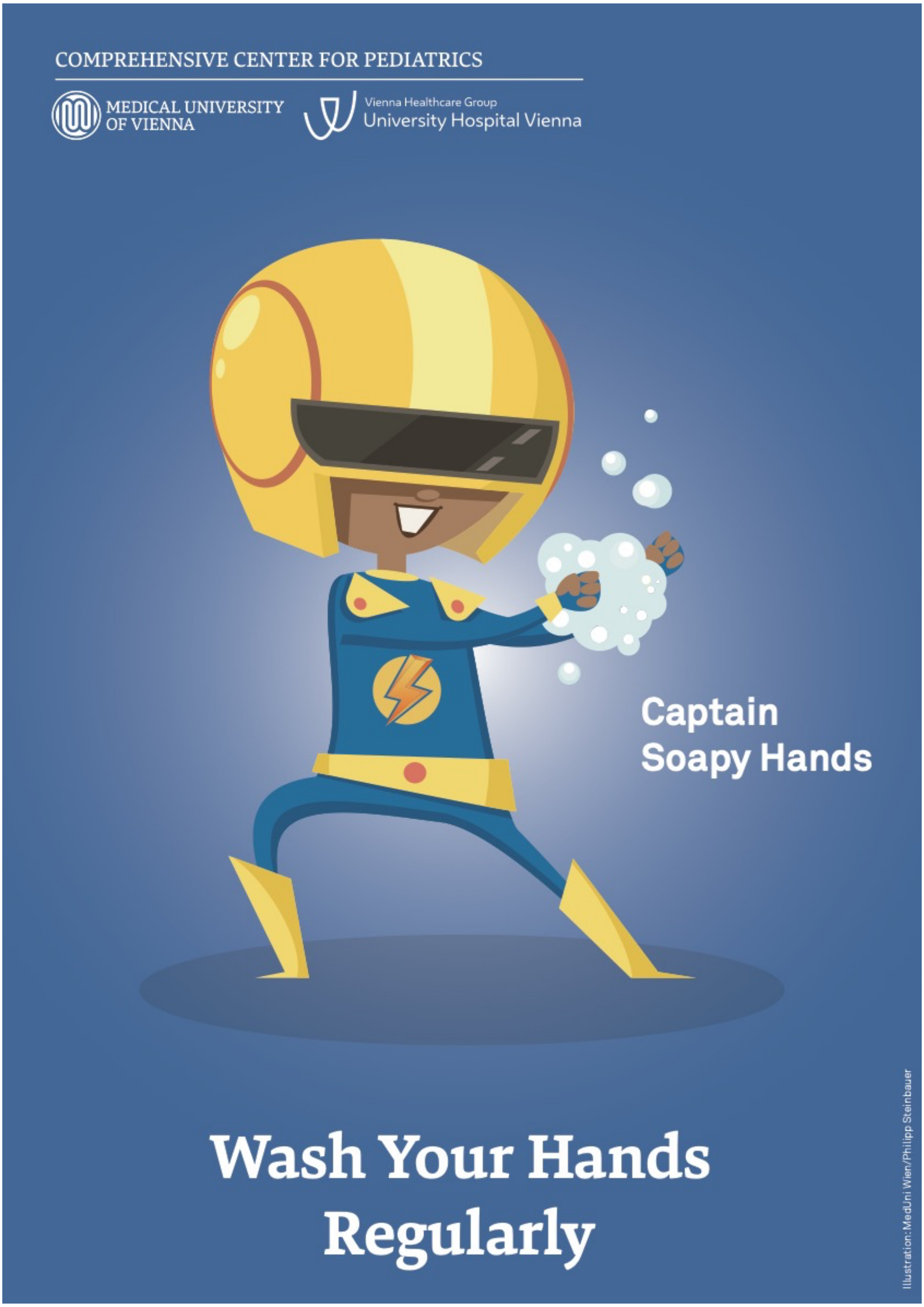

b. **Green Hero**

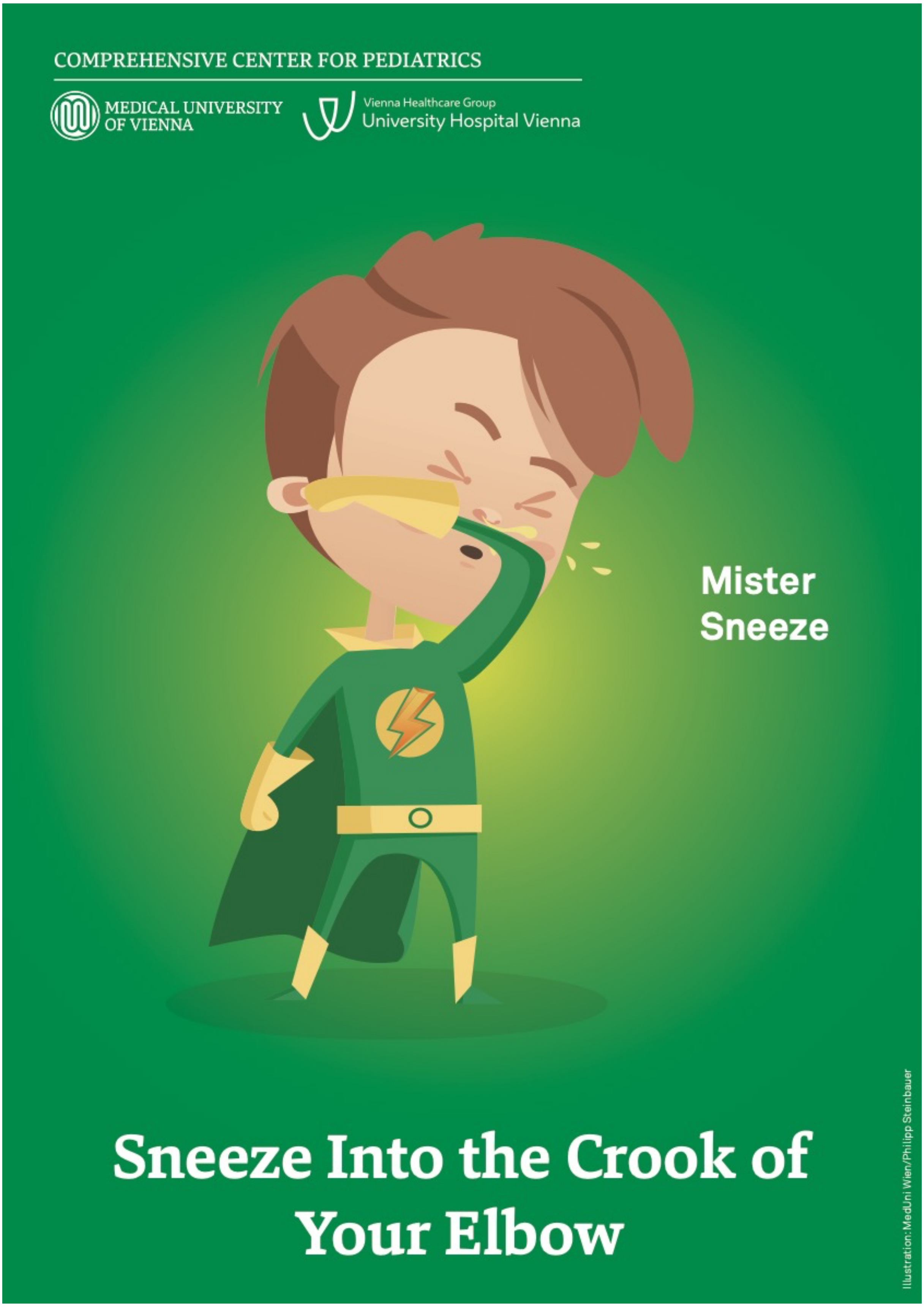
COMPREHENSIVE CENTER FOR PEDIATRICS
MEDICAL UNIVERSITY OF VIENNA
Vienna Healthcare Group
University Hospital Vienna
Mister Sneeze
Sneeze Into the Crook of Your Elbow
Illustration: MedUni Wien/Philipp Steinbauer

c. **Orange Hero**

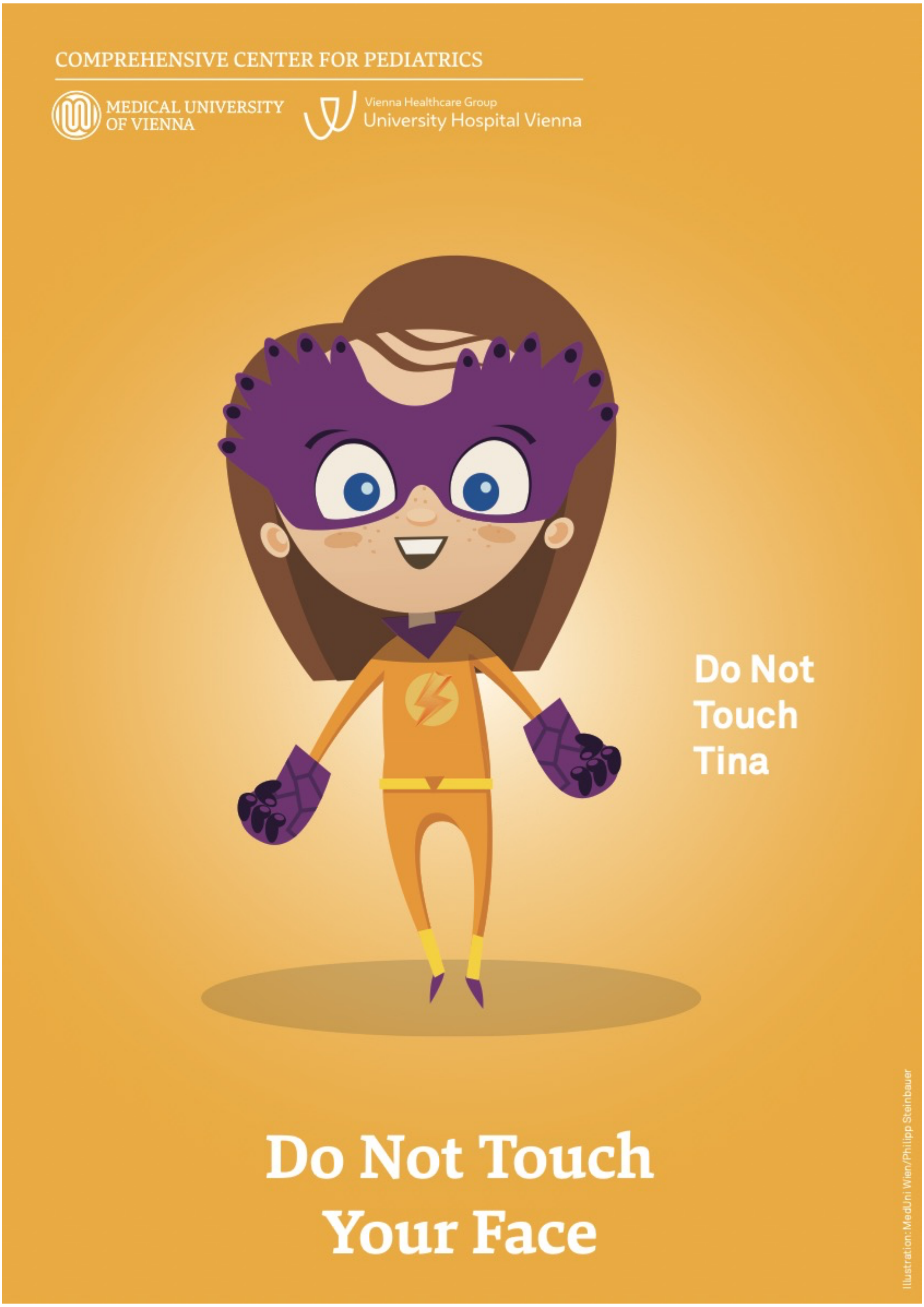

d. **Red Hero**

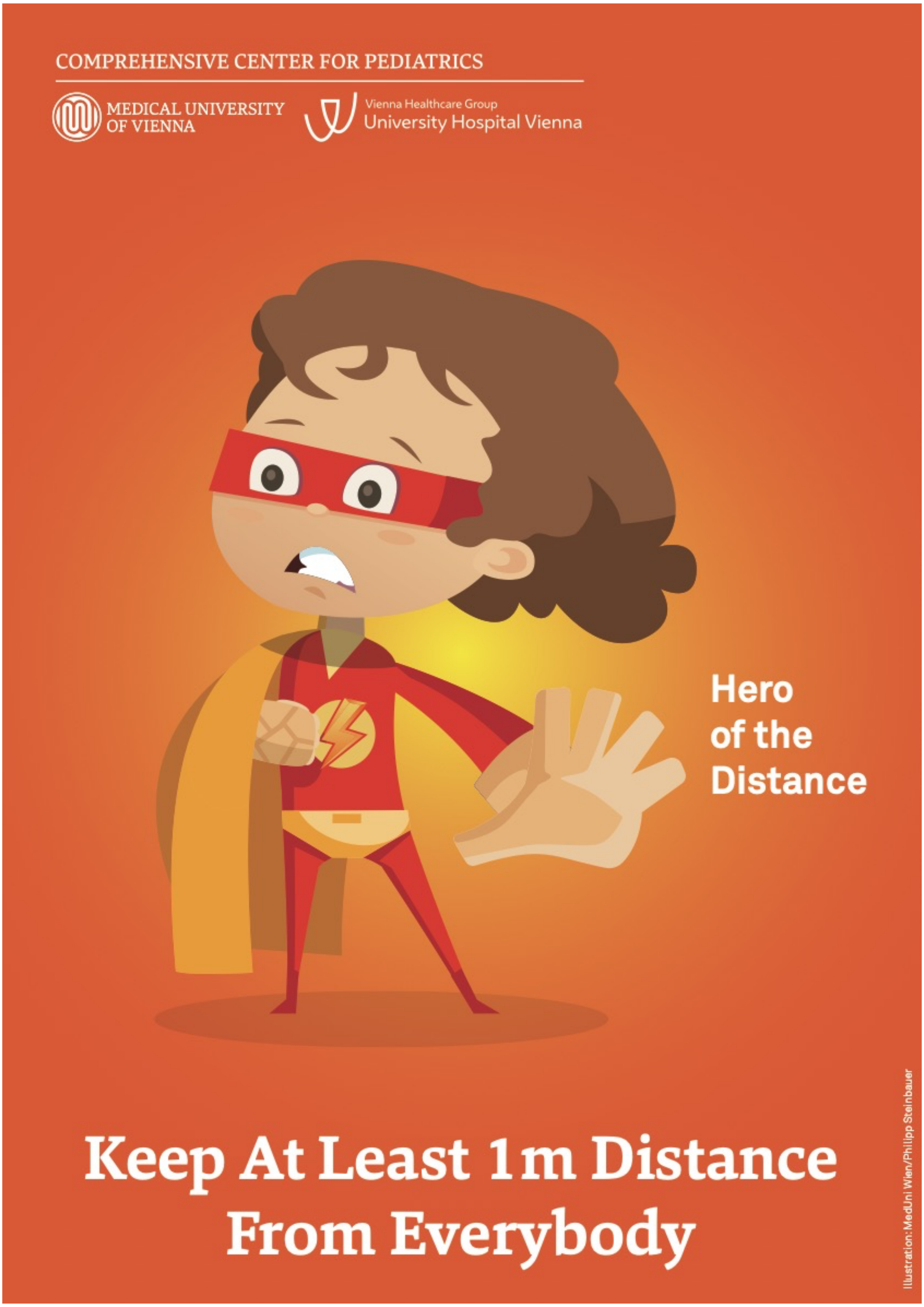
COMPREHENSIVE CENTER FOR PEDIATRICS
MEDICAL UNIVERSITY OF VIENNA
Vienna Healthcare Group
University Hospital Vienna
Hero of the Distance
Keep At Least 1m Distance From Everybody
Illustration: MedUni Wien/Philipp Steinbauer

e. **Pink Hero**

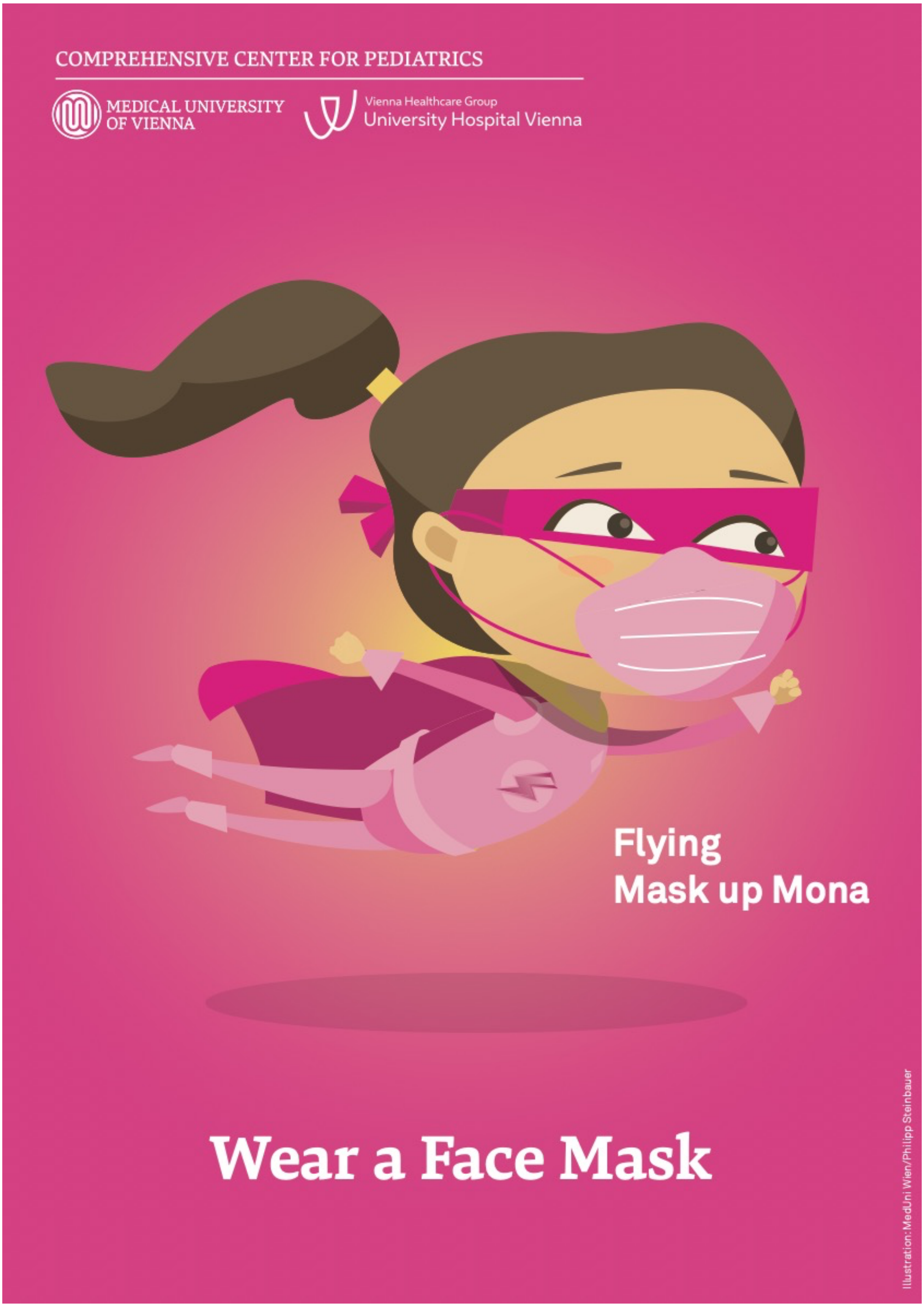
COMPREHENSIVE CENTER FOR PEDIATRICS
MEDICAL UNIVERSITY OF VIENNA
Vienna Healthcare Group
University Hospital Vienna
Flying
Mask up Mona
Wear a Face Mask
Illustration: MedUni Wien/Philipp Steinbauer

3. **Corona Quiz**

   a. **Question 1**

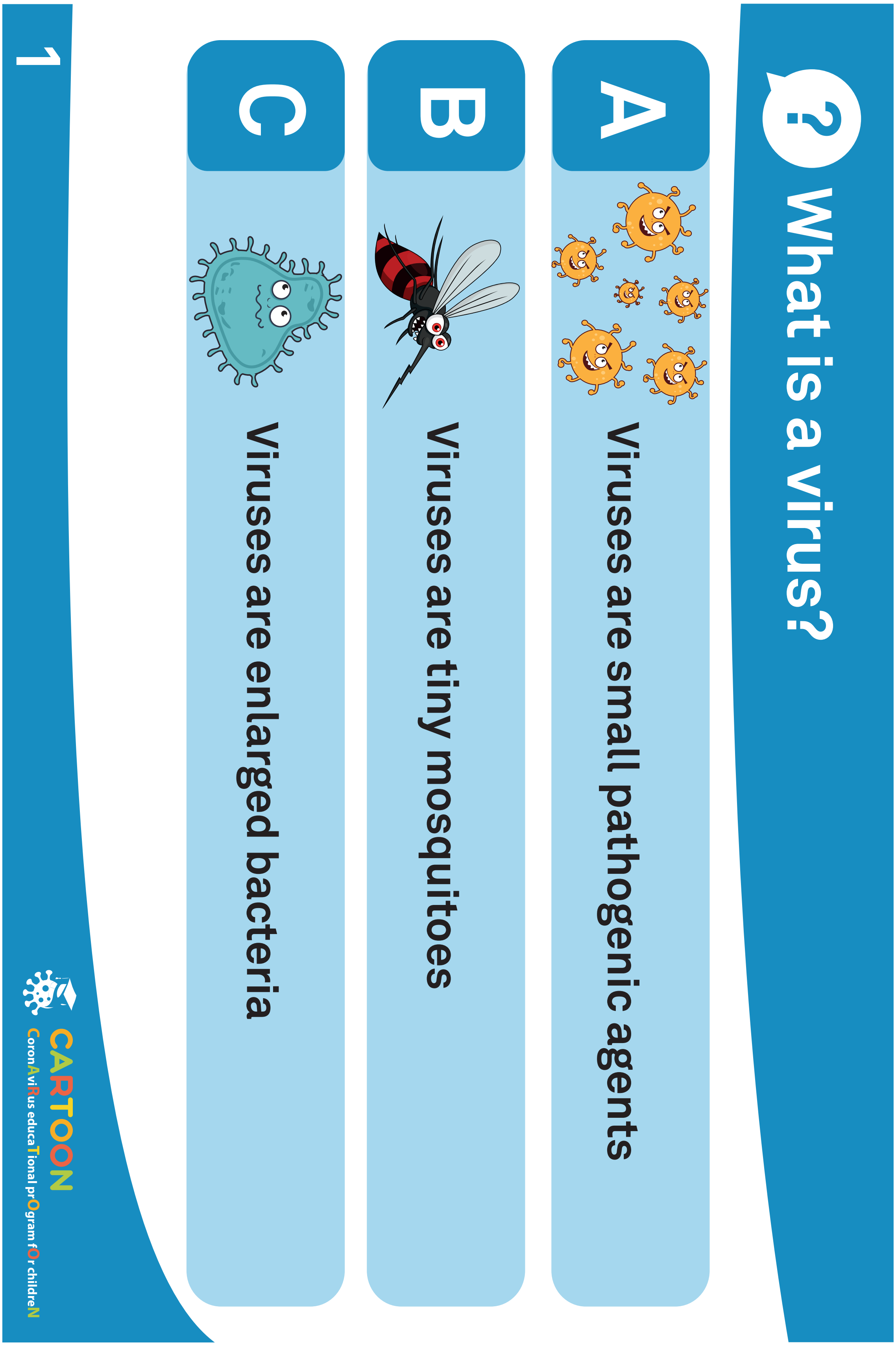

b. **Question 2**

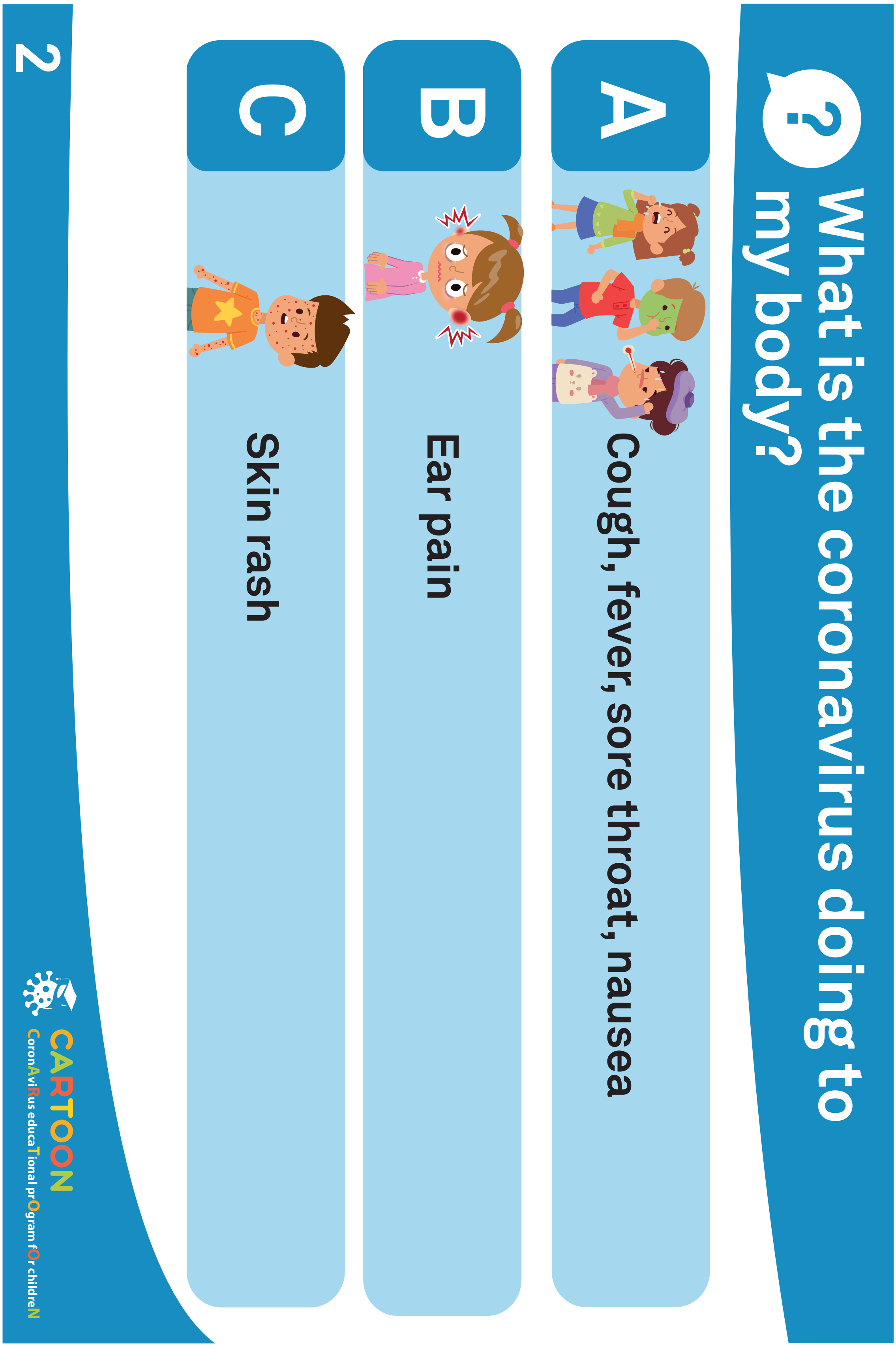

What is the coronavirus doing to my body?
A
Cough, fever, sore throat, nausea
B
Ear pain
C
Skin rash
2
CARTOON
CoronAviRus educaTional prOgram fOr childreN

c. **Question 3**

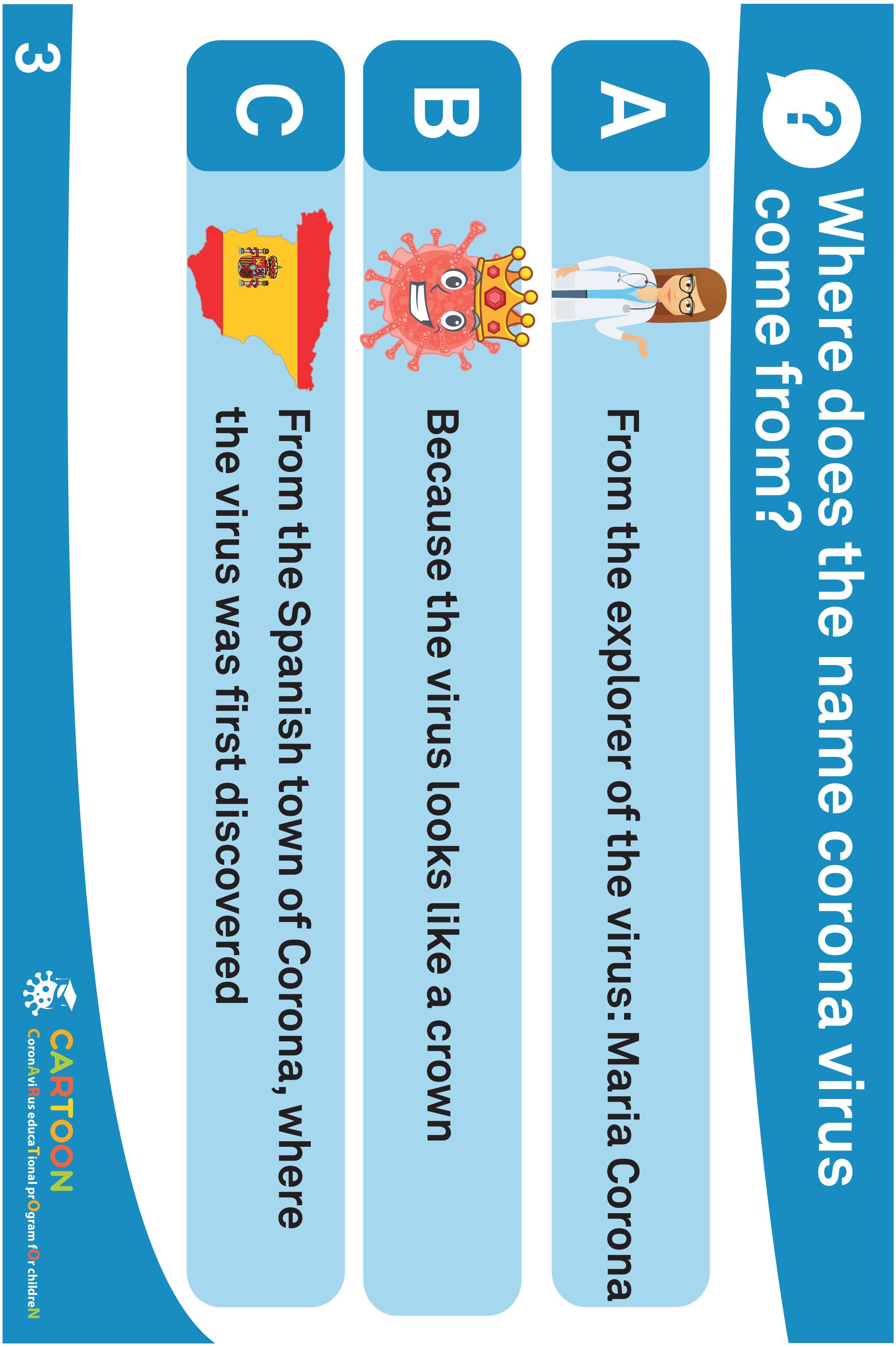
?
Where does the name corona virus come from?
A
From the explorer of the virus: Maria Corona
B
Because the virus looks like a crown
C
From the Spanish town of Corona, where the virus was first discovered
3
CARTOON
CoronAviRus educaTional prOgram fOr childreN

d. Question 4

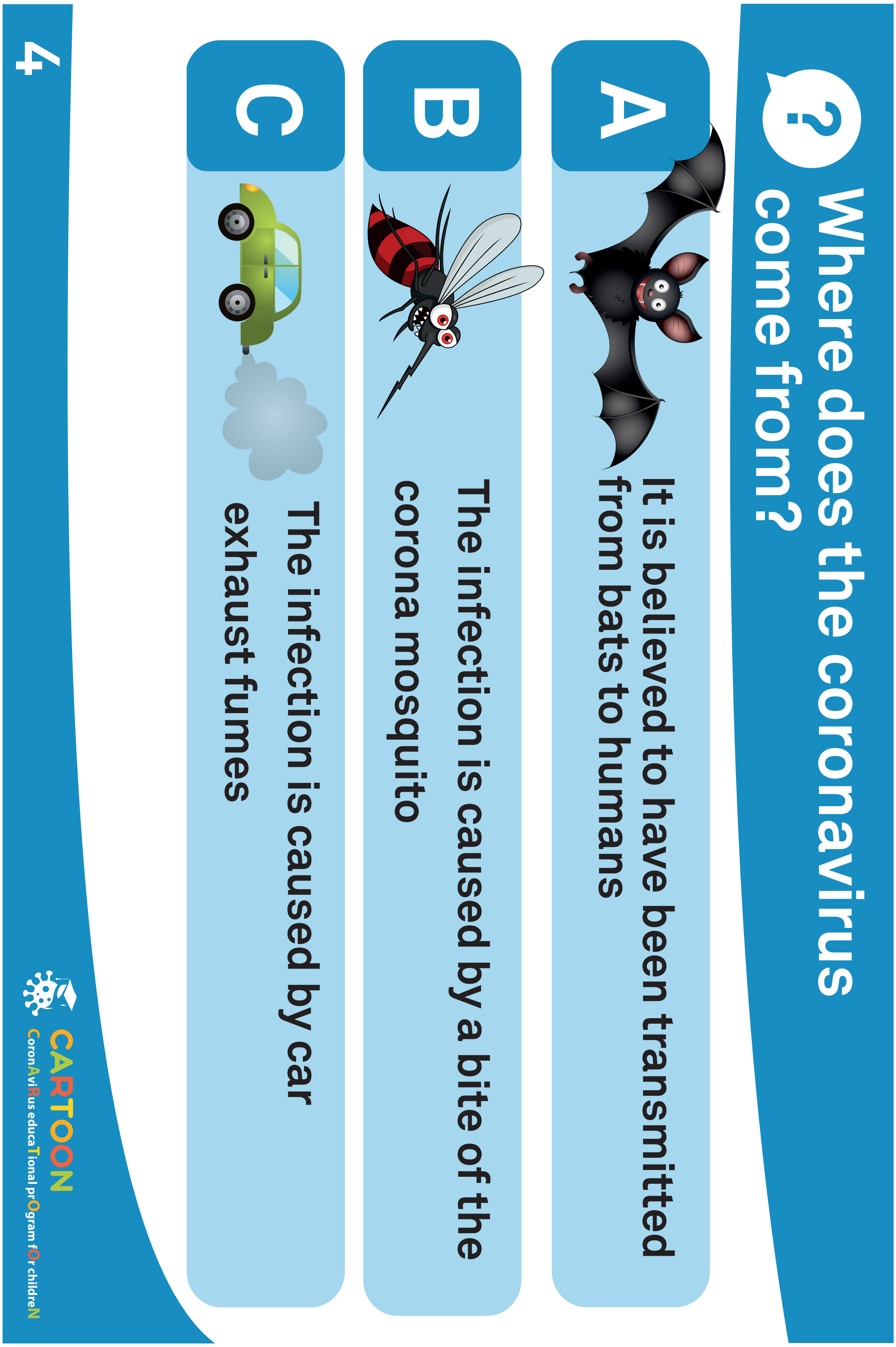

4
Where does the coronavirus come from?
A
It is believed to have been transmitted from bats to humans
B
The infection is caused by a bite of the corona mosquito
C
The infection is caused by car exhaust fumes
CARTOON
CoronAviRus educaTional prOgram fOr childreN

e. Question 5

How can you get infected with the coronavirus?

A

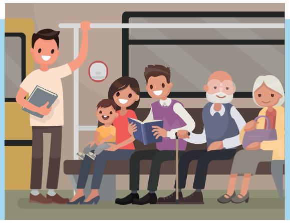

when you sit next to someone on the train

B

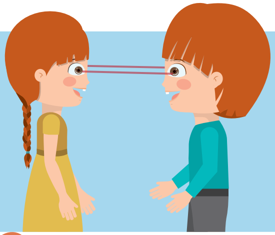

If you look into each other's eyes for too long

C

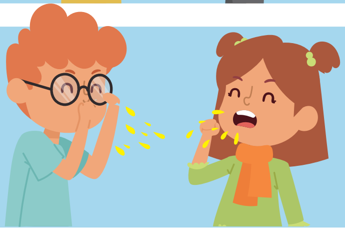

If you are coughed or sneezed on by someone who is infected with the virus

5

CARTOON
CoronAviRus educaTional prOgram fOr childreN

f. **Question 6**

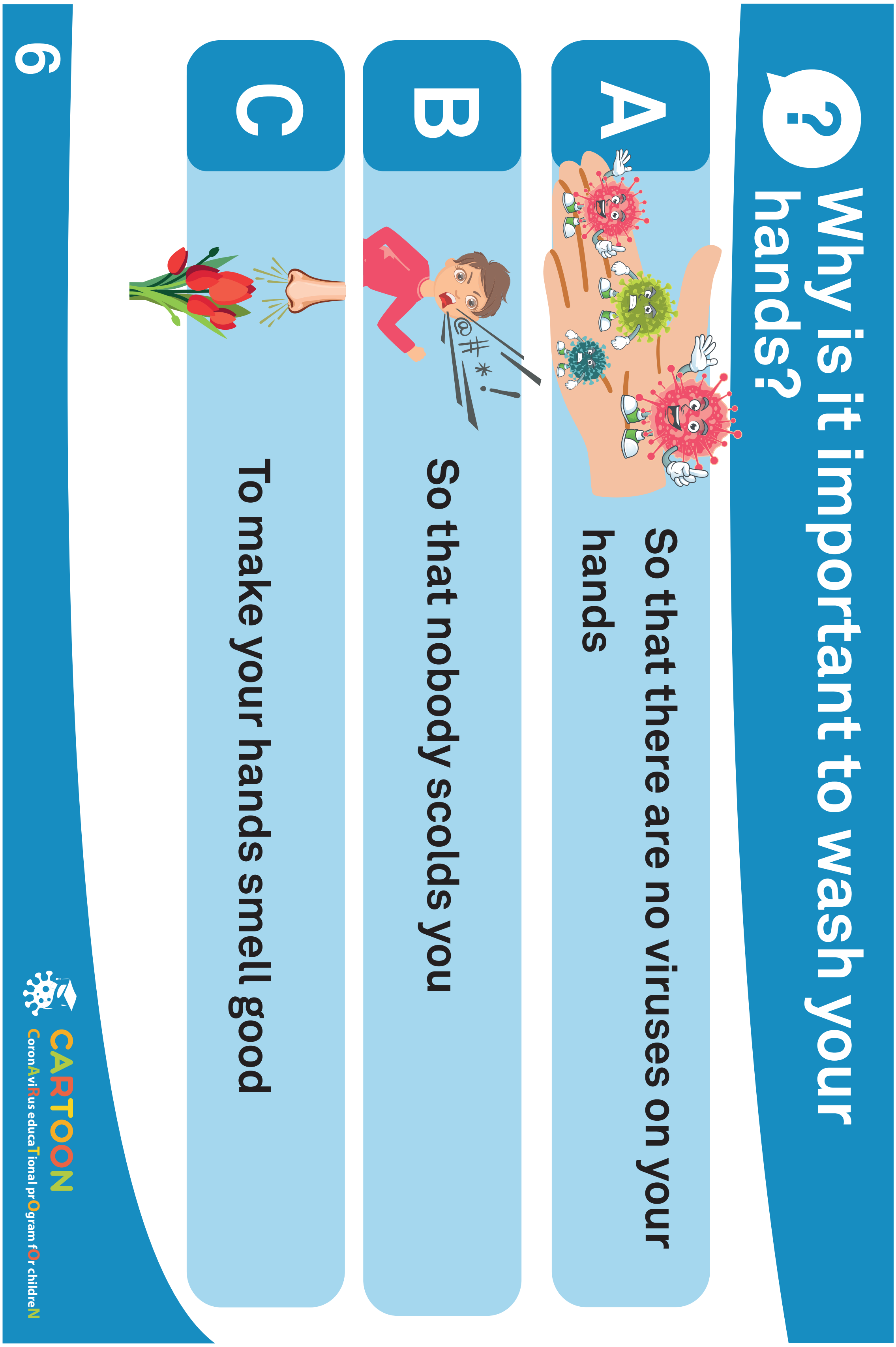

Why is it important to wash your hands?
A
So that there are no viruses on your hands
B
So that nobody scolds you
C
To make your hands smell good
6
CARTOON
CoronAviRus educaTional prOgram fOr childreN

g. Question 7

How to wash your hands correctly?

A

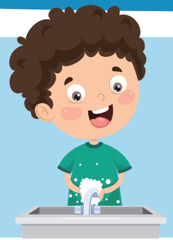

I wash the complete hands - with the back of the hand, between the fingers and under the nails

B

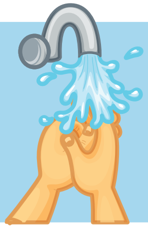

Allow water to run over it briefly

C

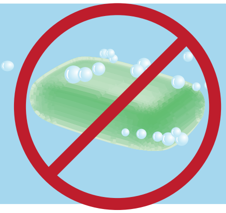

Never use soap, because coronaviruses love soap

7

CARTOON
CoronAviRus educaTional prOgram fOr childreN

h. Question 8

How long should you wash your hands?

A 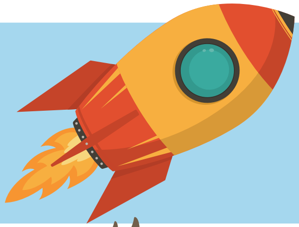 As fast as possible

B 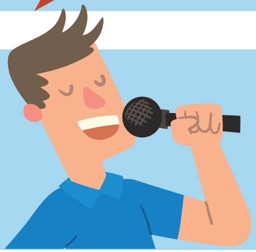 I sing „happy birthday“ two times

C 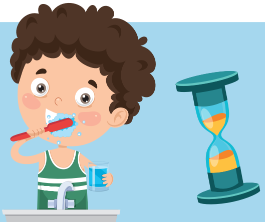 As long as I am brushing my teeth

8

CARTOON
CoronAviRus educaTional prOgram fOr childreN

i. **Question 9**

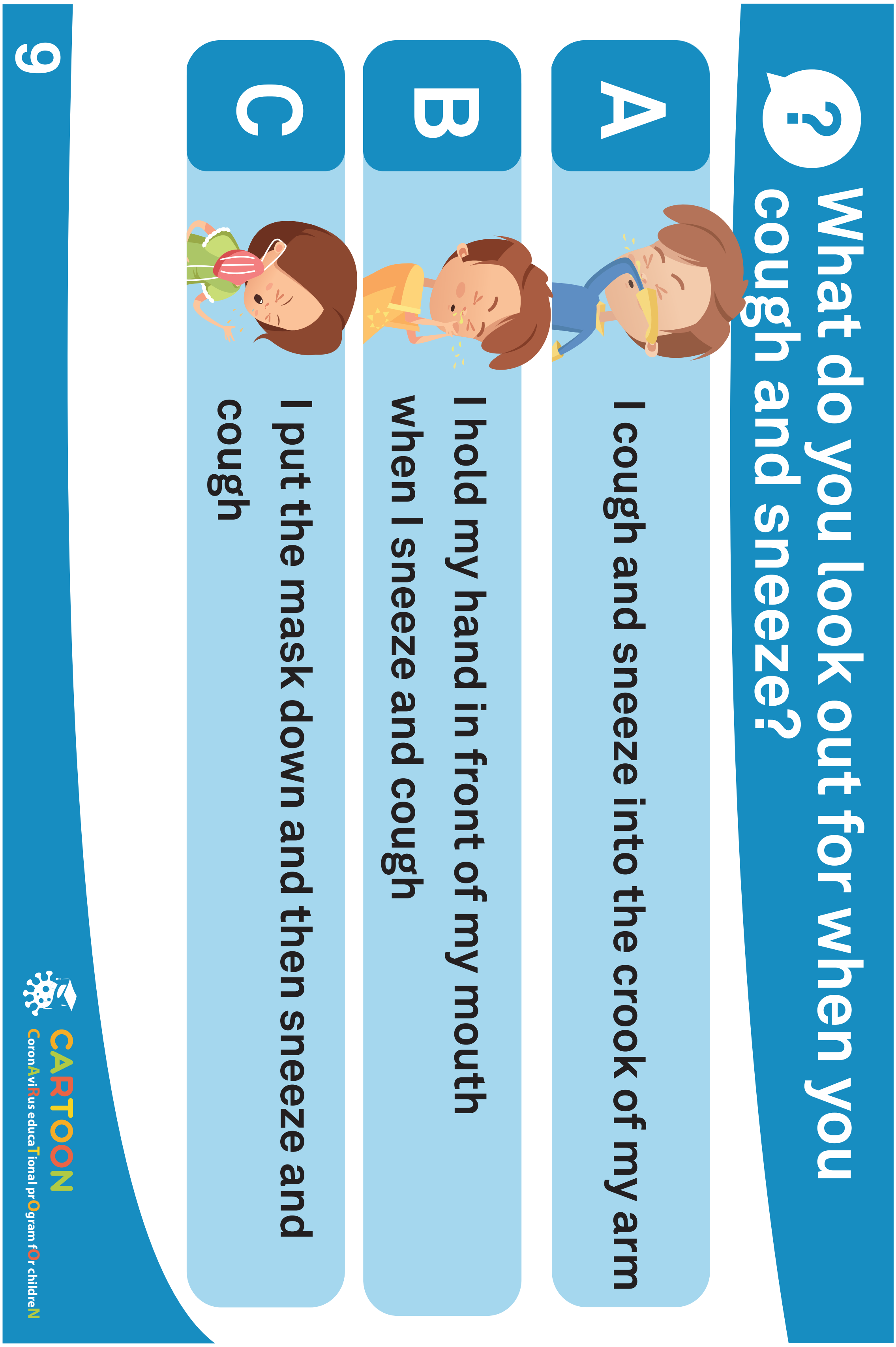
9
?
What do you look out for when you cough and sneeze?
A
I cough and sneeze into the crook of my arm
B
I hold my hand in front of my mouth when I sneeze and cough
C
I put the mask down and then sneeze and cough
CARTOON
CoronAviRus educaTional prOgram fOr childreN

j. Question 10

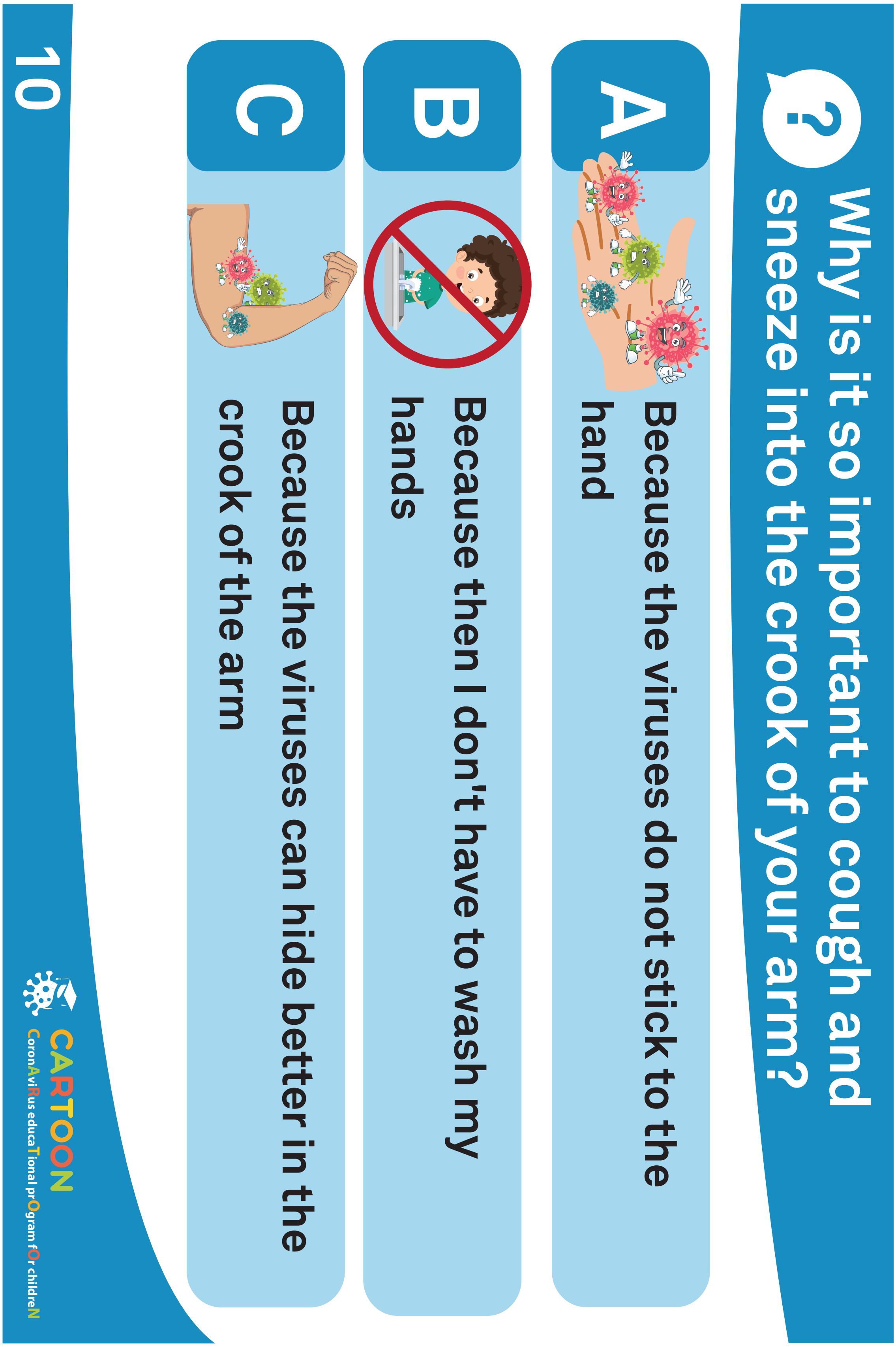

k. Question 11

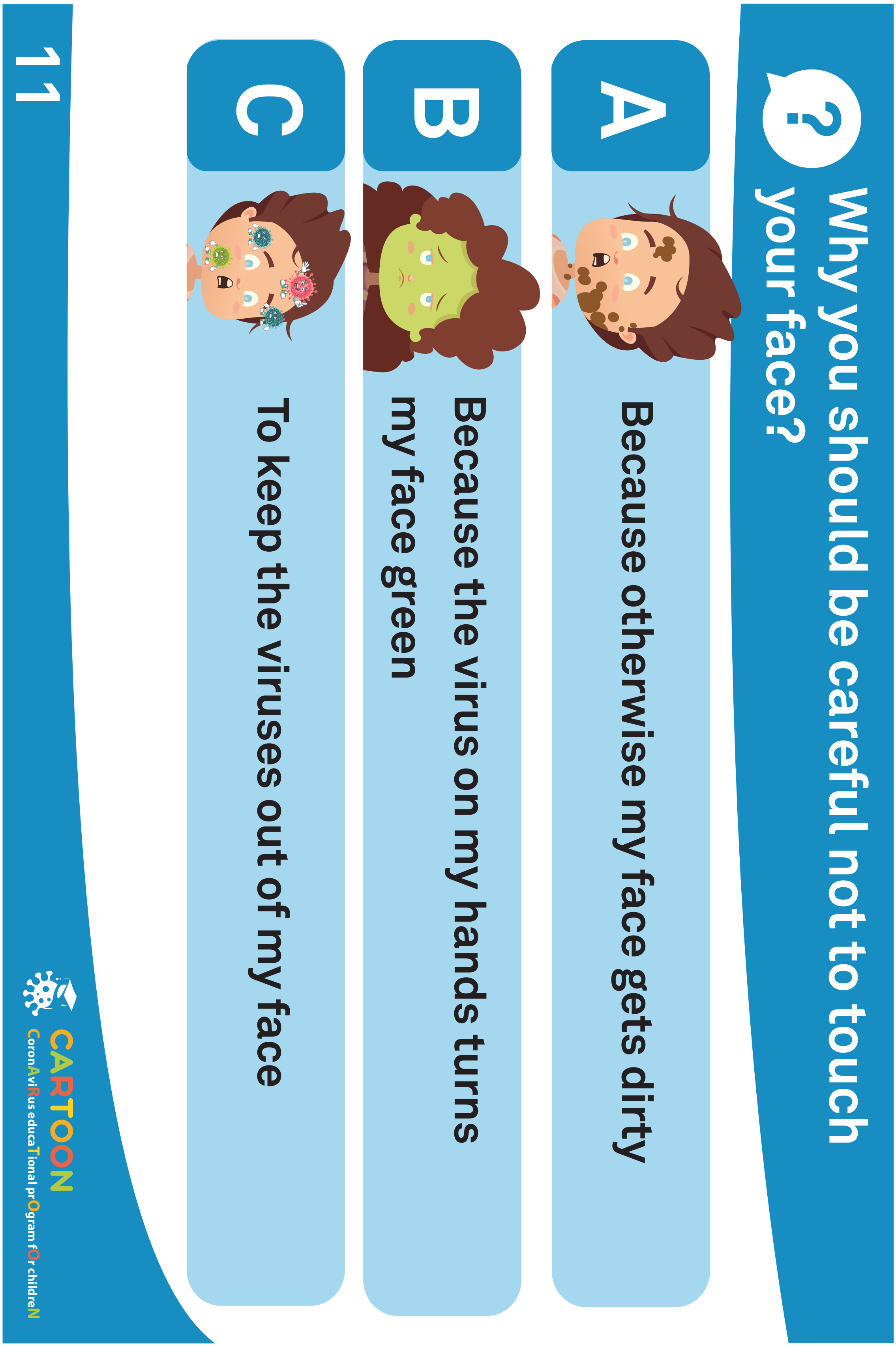

l. **Question 12**

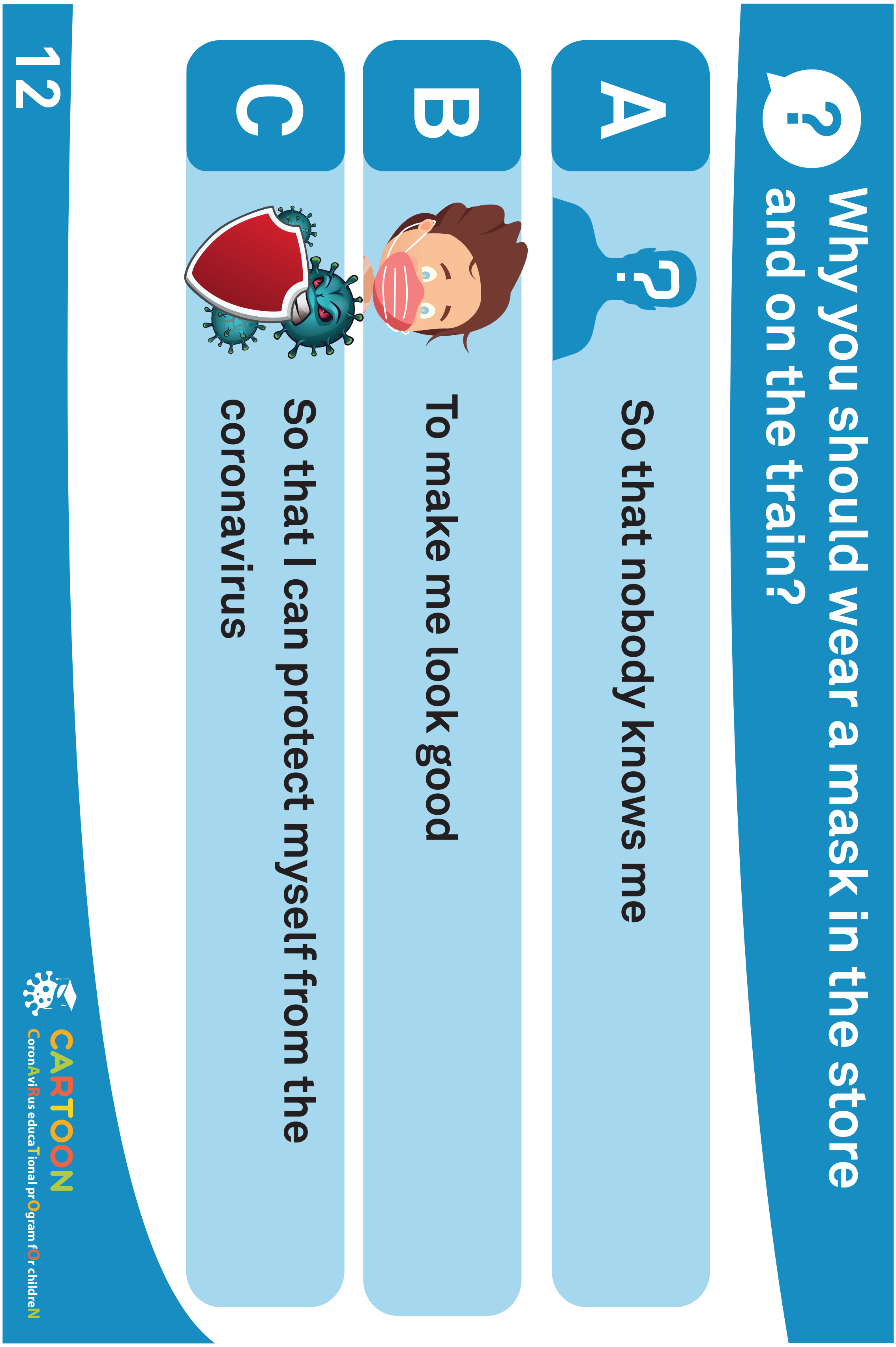

m. Question 13

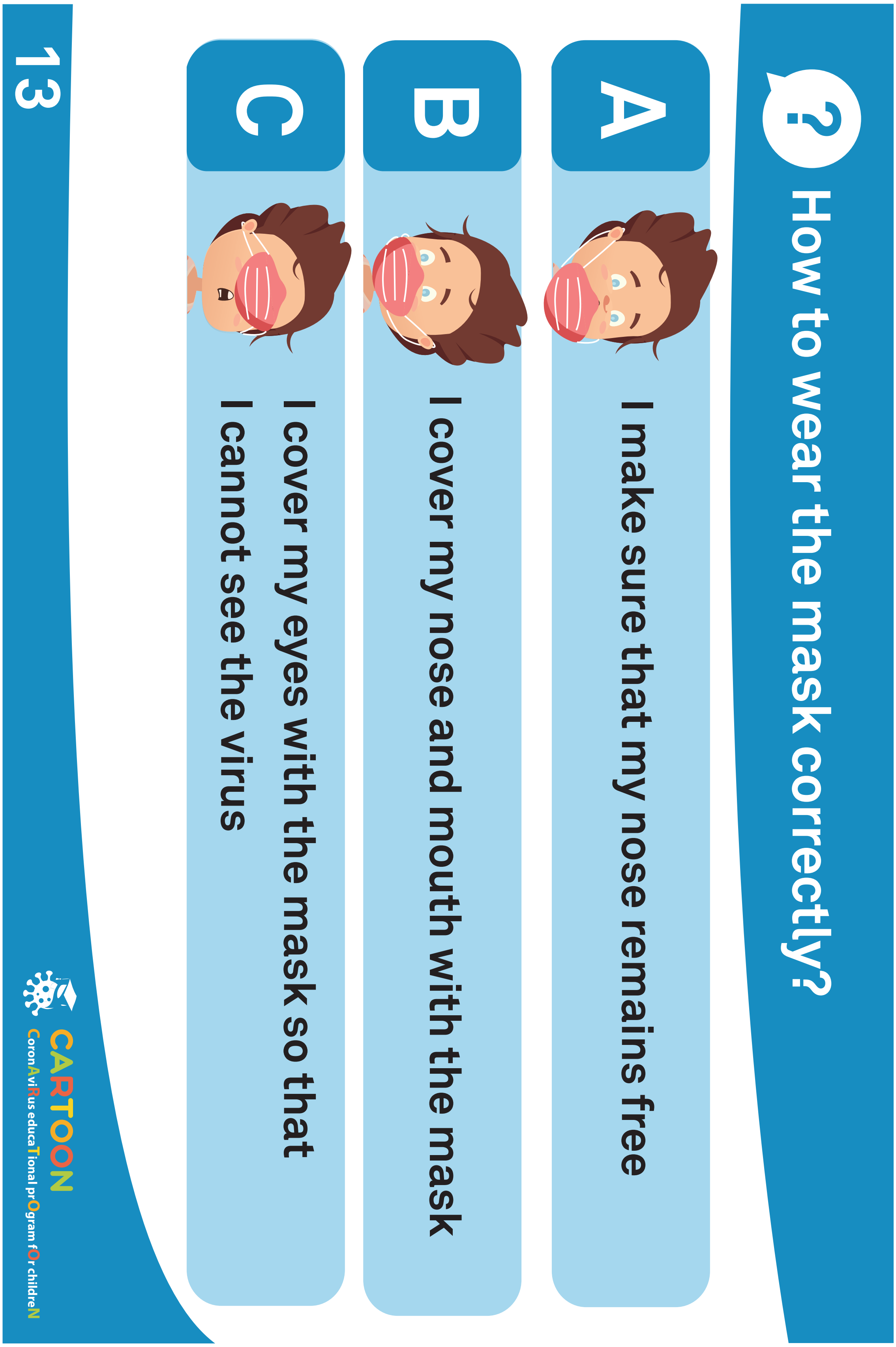

n. **Question 14**

Why is it important to keep your distance from other people?

A 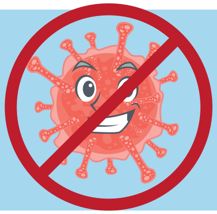 To better protect myself against the virus

B 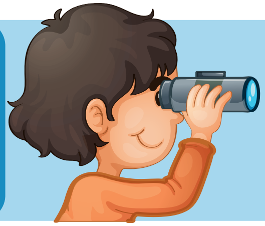 So that I can observe other people better

C 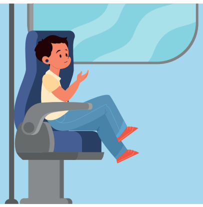 So that I have more space

14

CARTOON
CoronAviRus educaTional prOgram fOr childreN

o. **Question 15**

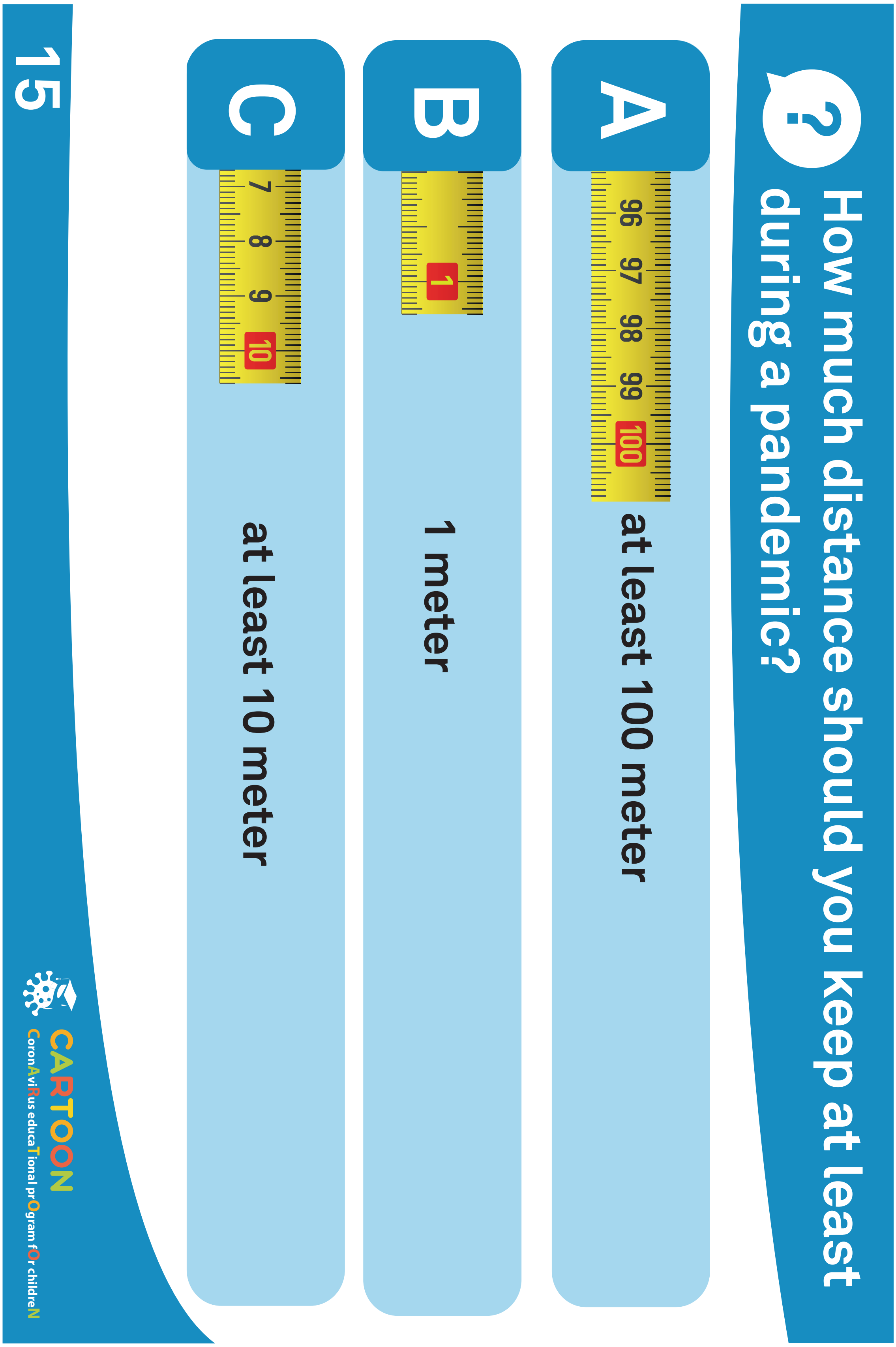

p. Question 16

What is a pandemic?

A 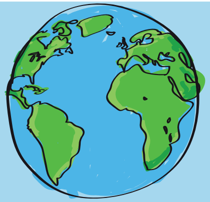 The whole world is affected by the same virus

B 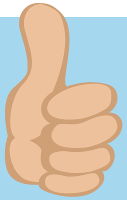 That is a cooler word for „virus“

C 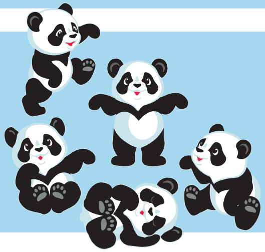 A meeting of many panda bears

16

CARTOON
CoronAviRus educaTional prOgram fOr childreN

q. Question 17

? What do you need a corona test for?

A 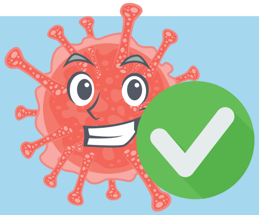 To know, if someone is infected with the coronavirus

B 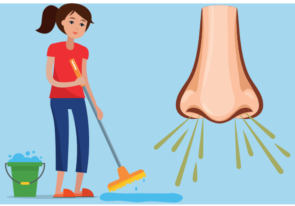 To blow my nose

C 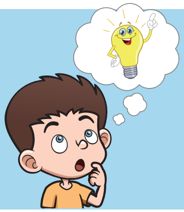 To check what special skills the virus has

CARTOON
CoronAviRus educaTional prOgram fOr childreN

r. Question 18

Who is doing the corona test?

A 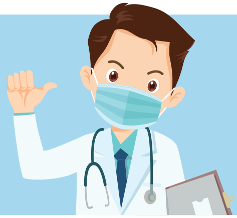 A doctor or nurse

B 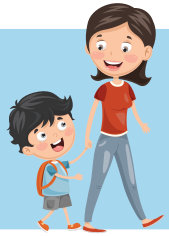 My mum

C 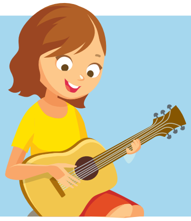 The nursery school teacher

18

CARTOON
CoronAviRus educaTional prOgram fOr childreN
